# Dose‐Dependent Effects of Catecholaminergic Modulation on Interference Control: Role of Baseline GABA and Glx in Cortico‐Subcortical Networks

**DOI:** 10.1002/hbm.70385

**Published:** 2025-10-23

**Authors:** Anna Helin Koyun, Annett Werner, Paul Kuntke, Veit Roessner, Christian Beste, Ann‐Kathrin Stock

**Affiliations:** ^1^ Cognitive Neurophysiology, Department of Child and Adolescent Psychiatry, Faculty of Medicine TU Dresden Dresden Germany; ^2^ University Neuropsychology Center, Faculty of Medicine TU Dresden Dresden Germany; ^3^ Institute of Diagnostic and Interventional Neuroradiology TU Dresden Dresden Germany; ^4^ German Center for Child and Adolescent Health (DZKJ), Partner Site Leipzig/Dresden Dresden Germany

**Keywords:** catecholamines, GABA+, Glx, interference control, MRS

## Abstract

Cognitive control, which is critical for goal‐directed behavior, involves resolving conflicts between competing stimuli and is influenced by neurotransmitter interactions within cortico‐subcortical areas. This study investigated the relationship between baseline amino acid transmitter levels and interference control, focusing on the effects of experimentally enhancing catecholaminergic signaling. Using a double‐blind, placebo‐controlled crossover design with two dosage groups, *n* = 71 healthy human adults underwent proton magnetic resonance spectroscopy once to assess baseline GABA+ and Glx levels in the anterior cingulate cortex (ACC), striatum, and supplementary motor area (SMA). Participants then performed a subliminally primed flanker task inducing different scales of conflict twice while EEG was recorded: once after receiving a placebo (lactase) and once more under either low (0.25 mg/kg) or medium (0.50 mg/kg) doses of methylphenidate (MPH), which modulates the catecholaminergic and amino acid transmitter systems driving cognitive and interference control. Medium MPH doses were more effective than low doses at reducing subliminal interference effects, highlighting dose‐specific behavioral improvements. Higher striatal GABA+ levels led to better interference control at low doses, while lower ACC GABA+ and GABA+/Glx levels were associated with better interference control at medium doses, suggesting a dose‐dependent shift from striatal to ACC dominance in conflict resolution. Neurophysiological (EEG data) analyses revealed increased theta‐band (TBA) and alpha‐band activity (ABA) overlapping in the mid‐superior‐frontal and inferior‐frontal clusters under conditions of heightened cognitive control demands. The findings highlight that whether and how amino acid transmitter levels in cognitive control‐relevant regions modulate interference conflicts depends on the degree of catecholaminergic signaling.

## Graphical abstract

We found medium methylphenidate doses (0.5 mg/kg) to be more effective than low doses (0.25 mg/kg) at reducing subliminal interference effects, with higher striatal GABA+ levels leading to better interference control at low doses, while lower ACC GABA+ and GABA+/Glx levels led to better interference control at medium doses.

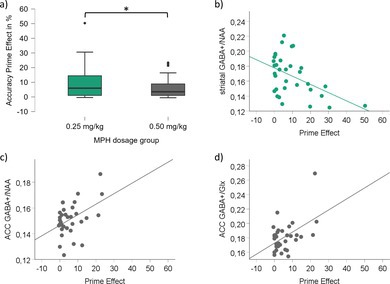

**Summary**

- We investigated the functional relationship between basic amino acid transmitter levels and catecholaminergic signaling with respect to interference control.
- Basic amino acid transmitter levels were assessed via MR spectroscopy, catecholaminergic signaling was experimentally manipulated with methylphenidate (MPH) (0.25 mg/kg or 0.50 mg/kg vs. placebo) and interference control was assessed with a subliminally primed flanker task.
- Medium MPH doses were more effective than low doses at reducing subliminal interference effects, with higher striatal GABA+ levels leading to better interference control at low MPH doses, while lower ACC GABA+ and GABA+/Glx levels led to better interference control at medium doses, thus suggesting a dose‐dependent shift from striatal to ACC dominance in conflict resolution.

## 1 Introduction

Recent advances in cognitive neuroscience have demonstrated the crucial role of amino acid and catecholaminergic neurotransmitters for cognitive control. Yet, the precise mechanisms by which these neurotransmitters interact and regulate how the brain resolves interference from competing or distracting stimuli remain to be explored. With respect to the most important brain regions underlying cognitive control, it is well known that the striatum and the anterior cingulate cortex (ACC) drive the selection of appropriate actions (Adams et al. 2017; Redgrave et al. 2011), while the supplemental motor area (SMA) and ACC play an important role in the activation and suppression of pre‐potent responses (Bari and Robbins 2013) to optimize interference control.

The intricate interplay between GABAergic inhibition and glutamatergic excitation within these areas is likely key for optimizing goal‐directed cognitive and behavioral adjustments (Quetscher et al. 2015; Silveri et al. 2013; Takei et al. 2016). While findings may vary depending on the investigated sample and context, it can generally be assumed that (GABAergic) lateral inhibition within the striatum as well as (glutamatergic) activation/excitation in the ACC and SMA should be beneficial for interference control (Bar‐Gad et al. 2003; Beste et al. 2008; Beste and Saft 2015; Botvinick et al. 2004; Boy, Evans, et al. 2010; Boy, Husain, et al. 2010; Haag et al. 2015; Yildiz et al. 2014). The reason for this lies in the winner‐takes‐all principles, which seem to govern the efficiency of processing in fronto‐striatal circuits (Bar‐Gad et al. 2003; Beste et al. 2018; Beste et al. 2014; Humphries et al. 2006; Plenz 2003). According to these principles, striatal GABAergic neurons support response selection by laterally inhibiting competing response options provided by glutamatergic input from the prefrontal cortex (PFC) and feeding the end result of this selection into connected cortical endpoints. Data obtained from patients with neurological disorders affecting the GABAergic‐glutamatergic interplay have been shown to support this assumption (Beste et al. 2014; Tomkins et al. 2013).

The functioning of the GABAergic‐glutamatergic interplay must be viewed in connection with catecholamines, which strongly modulate striatal functioning (Arnsten 2011). Catecholamines, especially dopamine (DA), are known to modulate the sensitivity of amino acid‐driven synaptic signaling in fronto‐striatal loops (Manz et al. 2021; Plenz 2003). Corroborating this, it has been shown that methylphenidate (MPH), which acts as a mixed DA and noradrenaline (NE) transporter blocker, not only increases extracellular levels of DA/NE in the striatum and prefrontal cortex (Dipasquale et al. 2020; Spencer et al. 2012) but also increases GABA release from striatal interneurons (Freese et al. 2012; Tritsch and Sabatini 2012), reduces glutamate levels in cortico‐subcortical areas, and affects PFC glutamate receptors dose‐dependently (Carrey et al. 2002; Cheng et al. 2014; Erlij et al. 2012; Urban et al. 2013). Overall, catecholamines are known to facilitate conflict monitoring and the selection of appropriate responses among competing response options (Bensmann et al. 2020; Mückschel, Gohil, et al. 2017; Willemssen et al. 2011), but findings may vary depending on the investigated sample and context. Given the common agreement on an underlying inverted u‐shaped curve (with optimal performance at medium levels of catecholamine release), it can be generally assumed that a slight to moderate increase in catecholaminergic signaling should be beneficial for interference control, while a strong increase might worsen performance towards the downward end of the inverted u‐shaped curve (Arnsten 2011; Bar‐Gad et al. 2003; Bensmann, Zink, Arning, et al. 2019; Bensmann et al. 2020; Petzold et al. 2019; Robbins and Arnsten 2009).

To this day, however, it has remained largely unclear how amino acid transmitters and catecholamines interact in facilitating or hindering interference control in human subjects. Clarifying this intricate interplay could provide a valuable framework for understanding the neural mechanisms underlying cognitive and interference control, help explain inter‐individual differences, and potentially also help to optimize this faculty in those reporting/displaying poor performance. To address this nexus, our current study took a multi‐modal approach: Magnetic resonance spectroscopy (MRS) was used to assess baseline variations in GABA+ (GABA plus macromolecules) and Glx (glutamate + glutamine) in cognitive control‐relevant regions (i.e., the ACC, SMA, and the striatum). Additionally, MPH was experimentally administered at low and medium doses (relative to the recommended maximum dose). High doses were omitted as they are more likely to induce a marked performance‐impairing over‐stimulation along the aforementioned inverted u‐shaped curve (Bensmann, Zink, Roessner, et al. 2019; Linssen et al. 2014; Rostron et al. 2013). Interference control was investigated with a primed flanker task (Bensmann, Zink, Roessner, et al. 2019; Stock et al. 2016). To investigate the neurophysiological processes and neuronal sources underlying baseline and modulation‐induced differences in interference control, we assessed (frontocentral) theta and alpha oscillations (Dockree et al. 2017; Prochnow et al. 2024). Theta band activity (TBA), particularly in the SMA and medial frontal cortex, is linked to conflict detection and resolution, while alpha band activity (ABA) facilitates the inhibition of irrelevant stimuli and maintaining task focus (Beste et al. 2023; Cavanagh and Frank 2014; Klimesch 2012).

We expected that participants receiving medium MPH doses would exhibit smaller behavioral interference effects than those receiving lower doses (Arnsten 2011; Bar‐Gad et al. 2003; Bensmann et al. 2020; Bensmann, Zink, Arning, et al. 2019; Petzold et al. 2019; Robbins and Arnsten 2009). Based on previous studies, we further expected that MPH should increase TBA and likely also alter ABA (Berchou et al. 1986; Farias et al. 2019; Hodzhev et al. 2012; Koyun, Wendiggensen, et al. 2024), with potentially larger effects at medium (as compared to low) MPH doses. MPH dose‐dependent ABA and TBA modulations should be reflected in control‐relevant brain areas including the investigated ones (i.e., the ACC, SMA, and striatum) (Adams et al. 2017; Bari and Robbins 2013; Redgrave et al. 2011). We further hypothesized that more neuronal inhibition (i.e., lower Glx or higher GABA+ levels) in the striatum as well as more neuronal excitation (i.e., higher Glx or lower GABA+ levels) in the ACC (and to a lesser extent possibly also within the SMA) would likely lead to better interference control and differentially predict MPH dose‐dependent effects on response and interference control (Bar‐Gad et al. 2003; Beste et al. 2008; Beste and Saft 2015; Botvinick et al. 2004; Boy, Evans, et al. 2010; Boy, Husain, et al. 2010; Haag et al. 2015; Yildiz et al. 2014). As we were interested in the effects of variations within each neurotransmitter system, the experimental MPH dose variation and the natural baseline variation in MRS‐assessed transmitters were key to analyzing our research question.

## 2 Materials and Methods

### 2.1 Participants

An initial priori estimation of the sample size using GPower software (Faul et al. 2007) yielded a required sample size of *n* = 80 (for an ANOVA within–between interaction of 2 × 2 groups and 2 × 2 measurements for *α* = 0.05, 1 − *β* = 0.95 and an estimated *f* = 0.20). Of note, a sample size of *n* = 78 recruited and *n* = 64 analyzed individuals had previously yielded significant results in a study with a comparable experimental design (both in terms of ANOVA interaction effects and correlation/regression effects of MRS‐assessed data) (Koyun et al. 2025).

*N* = 84 young and healthy adults (mean age 25.25 years ± 0.319 SEM; 37 females) voluntarily participated in this study. All participants were between 20 and 31 years old, had normal or corrected‐to‐normal vision, had no current/reported history of psychiatric, neurologic, or developmental disorders, reported no CNS‐affecting medication use, were neither pregnant nor breastfeeding, and fulfilled all requirements for MRI compatibility. Participants provided written informed consent and received financial compensation or course credits for their participation. The study was approved by the ethics committee of TU Dresden (EK 420092015) and conducted following the Declaration of Helsinki (Rickham 1964) and its later amendments. *N* = 8 participants were excluded from all subsequent analyses (behavioral, neurophysiological, and MRS data) due to the following reasons: *n* = 1 participant dropped out after the first appointment, *n* = 1 participant's response accuracy was below chance level (< 50%) in at least one experimental condition, *n* = 1 participant's response time was marked as an extreme outlier, and *n* = 5 were excluded due to technical problems during data recording. This resulted in a final sample of *n* = 76 participants (low dose MPH group: *n* = 38, mean age 25.10 ± 0.488, 18 females; medium dose MPH group: *n* = 38, mean age 25.31 ± 0.492, 15 females) included in the subsequent behavioral and neurophysiological data analyses. As for the MRS data analyses, *n* = 5 participants had to be additionally excluded due to poor shimming quality/SNR. Consequently, *n* = 71 participants (low dose MPH group: *n* = 35, mean age 24.83 ± 0.489, 16 females; medium dose MPH group: *n* = 36, mean age 25.47 ± 0.503, 13 females) were included in the linear correlation analyses.

### 2.2 Experimental Design and Methylphenidate Administration

A double‐blind MPH/placebo crossover design was used. The study involved one baseline MR‐spectroscopy measurement (at rest) due to the good test–retest reliability of MRS measures of the investigated metabolites (Baeshen et al. 2020; Duda et al. 2021; Shungu et al. 2016) and two experimental EEG sessions spaced 7 days apart. Pseudo‐randomization, as defined by the between‐subject factors of MPH dosage (low vs. medium dosage) and order of drug administration (MPH on the first appointment vs. MPH on the second appointment), established balanced sex ratios within and between each subgroup. Otherwise, the group assignment was random and double‐blind (i.e., neither the participants nor the experimenter knew the dosage group of any given participant or at which appointment placebo and MPH were administered). On one of the appointments, participants received the respective MPH dose (low: 0.25 mg, medium: 0.50 mg per kg body weight) and a lactose placebo on the other in a double‐blind fashion. The doses were labelled as “low” and “medium” based on the maximum recommended dose of 0.8 mg/kg. The experiment began approximately 2 h after MPH/placebo administration, aligning with peak MPH plasma levels, which typically occur between 1 and 3 h, with the maximum drug concentration usually reached around 2 h after oral administration (Challman and Lipsky 2000).

### 2.3 ^1^H‐MRS Data Acquisition and Processing

All experimental MRI and MRS data were acquired with a Siemens 3 T Prisma scanner (Siemens Healthineers, Erlangen, Germany) using a 32‐channel (receive‐only) head rf coil. The concentrations of GABA+ (γ‐aminobutyric acid and macromolecules), Glx (glutamate and glutamine), and total N‐acetyl aspartate (tNAA; NAA + *N*‐acetyl‐aspartyl‐glutamate) in the striatum, SMA, and ACC were examined using ^1^H‐MRS.

For exact voxel placements, structural images were obtained using a high‐resolution 3D T1‐weighted sagittal Magnetization Prepared Rapid Gradient Echo (MPRAGE) sequence (1 mm isovoxel, TE/TR/TI: 2.29 ms/2.3 s/0.9 s) and reconstructed. Using ^1^H‐MRS, brain metabolite (GABA+, Glx, tNAA) concentrations in the striatum, ACC, and SMA were quantified. For this, separate voxels of interest (VOIs) were individually positioned for each region (see Section 2.4). In addition to the inbuilt shim routine, manual shimming was performed for each VOI, further optimizing spectral resolution. The shimming criterion was a full width at half maximum (FWHM) value below 20 Hz for the unsuppressed water signal. According to the recommendations of Peek et al. (2023), to obtain GABA+ and Glx values, we ran a MEGA‐PRESS (Mescher‐Garwood point‐resolved spectroscopy) sequence (echo time TE/repetition time TR = 68/3000 ms, edit ON acquisitions = 128, edit OFF acquisitions = 128) developed by Edward J. Auerbach and Małgorzata Marjańska and provided by the University of Minnesota (Marjańska et al. 2013; Tremblay et al. 2014), based on a C2P license agreement with Siemens Healthineers AG Germany.

**FIGURE 1:**
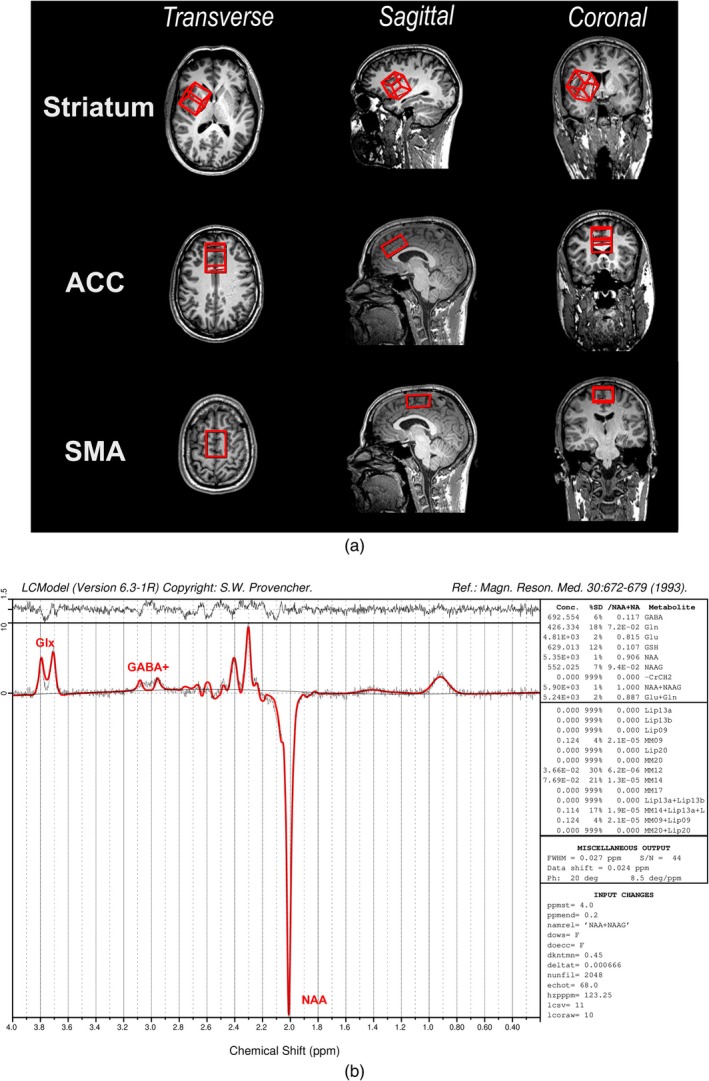
^1^H‐MRS voxel placement. (a) Depicted are the volumes of interest (VOIs) [from top to bottom]: In the striatum, the anterior cingulate cortex (ACC), and the (pre‐) supplementary motor areas (SMA). (b) A representative LCModel fit of MEGA‐PRESS for the ACC. Upper part: Residual curve (depicting the difference between the fitted and the measured curves). Lower part: Black curves represent the measured spectrum and the baseline. On the right are metabolite estimates (in arbitrary units). GABA+: GABA and macro molecules; Glx: combination of glutamate and glutamine; NAA: N‐Acetyl‐aspartate.

Finally, the ratios of GABA+ and Glx to tNAA in the obtained spectra were estimated. After exporting the data (“edit on,” “edit off,” svs_se30_ in *.rda format) directly from the spectroscopy subroutine of the scanner, difference spectra were calculated (using an in‐house Python script) and loaded into LCModel software (v6.3‐1H, copyright Stephen Provencher, Canada). A representative LCModel fit of MEGA‐PRESS can be found in Figure 1b. Basis sets for MEGA‐PRESS were delivered by Ulrike Dydak's Lab at Purdue University (https://www.purdue.edu/hhs/hsci/mrslab/basis_sets.html).

The “3T Siemens Difference Basis Set with Kaiser Coupling Constants,” was based on updated values for chemical shifts and J‐GABA coupling constants [Kaiser et al. 2008; Kreis and Bolliger 2012; Near et al. 2013; these slightly differ from the originally generated basis sets by Dydak et al. (2011), which used the values by Govindaraju et al. (2000)].

Based on the “edit off” spectra from the same MEGA‐PRESS measurement and using the corresponding “3T Siemens Edit‐off Basis set,” total N‐acetylaspartate (tNAA; NAA + *N*‐acetyl‐aspartyl‐glutamate) reference values for GABA+ and Glx were estimated. The spline baseline constraint (“DKNTMN”) within the LC‐model routine was adapted for the most reliable quantitation results. The DKNTMN parameter (minimum allowed spacing between spline knots) allows for flexibility in the baseline curve, possibly accounting for a significant portion of the variance in GABA+ and Glx levels and can result in underestimated values. Following a previously established procedure (Koyun, Talebi, et al. 2024; Stock et al. 2023), we optimized (testing within the range of 0.1–1.0) and finally adjusted the DKNTMN parameter to a value of 0.45 to minimize the measurement error of GABA+ and Glx (CRLB) without affecting the SNR. This approach was used for all three regions to ensure consistency with previous research and minimize potential measurement biases. To warrant adequate data quality, only spectra of final acceptable shim quality (FWHM of the tNAA peak of 3–7 Hz) were used for the subsequent quantification. The GABA+ error estimate was assessed in the sample, as this measure typically has a higher error than Glx or the reference metabolite. In doing so, values below the 15% Cramér‐Rao lower bound (CRLB or %SD) criterion were obtained for all three VOIs.

### 2.4 ^1^H‐MRS Voxel Placement and Data Processing

After the localizer, a high‐resolution 3D T1‐weighted sagittal MPRAGE sequence (1 mm is isovoxel) was measured and multiplanar reconstructed for exact voxel placements. Next, a 30 × 30 × 30 mm voxel of interest (VOI) was placed in the right striatum, and a 20 × 30 × 40 mm VOI was placed over the midline to cover large parts of both the left and right ACC (including only relatively small fractions of neighboring brain regions). Finally, a 20 × 30 × 40 mm VOI was positioned to cover the left and right (pre‐)SMA. Figure 1a illustrates the placement of the three VOIs.

Of note, the ^1^H‐MRS data reflects baseline levels of neurotransmitters, not variations related to experimental interventions or tasks. As Mikkelsen et al. (2017, 2019) recommended, the statistical analyses of the collected ^1^H‐MRS data utilized an internal metabolite reference signal. However, there must be no systematic association between the reference metabolite and the behavioral parameters under investigation (Mikkelsen et al. 2019). The absence of significant correlations between the behavioral measures (accuracy and response times) and tNAA (all *r* < 0.2; *p* > 0.1), justifies using tNAA (quantified from the edit‐off spectra) as a robust reference metabolite for GABA+ and Glx. A relative measure was formed by dividing GABA+/tNAA by Glx/tNAA to obtain a GABA+/Glx ratio (in which tNAA cancels out). The metrics derived for statistical evaluation were unitless ratios.

### 2.5 Experimental Task

**FIGURE 2:**
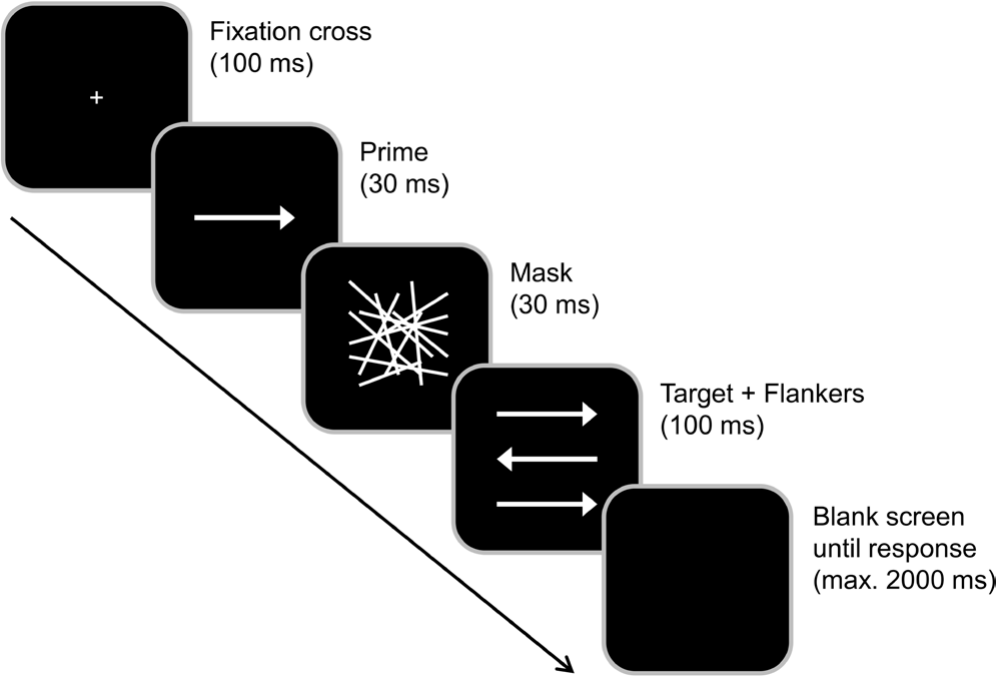
Experimental paradigm. Each trial started with a 100 ms presentation of a fixation cross, followed by a 30 ms presentation of a prime (middle arrow) and a 30 ms presentation of a mask (array). The target stimulus (middle arrow) and flankers were then simultaneously presented for 100 ms. After the presentation of the target, the screen turned black. Primes pointing in the same direction as the target were classified as compatible while flankers that pointed in the same direction as the target arrow were classified as congruent.

The task design and structure are described and illustrated in Figure 2. The experimental task was adapted from Boy, Evans, et al. (2010) and Boy, Husain, et al. (2010) and is identical to the paradigm utilized in previous studies (e.g., Bensmann et al. 2018; Stock et al. 2016). By combining the target stimulus with a subliminal prime and flankers, this task enables the examination of subconsciously and consciously evoked conflicts in response selection.

Participants were seated approximately 60 cm from a 24‐in. CRT monitor, which displayed stimuli against a black background. Manual responses were collected using the “Ctrl” buttons on a standard QWERTZ keyboard. “Presentation” software (Version 18.3 by Neurobehavioral Systems Inc.) managed stimulus presentation, response recording, and EEG synchronization. Each trial began with a central white fixation cross for 100 ms, followed by a subliminal prime (a white arrow pointing left or right) displayed centrally for 30 ms. Immediately after the prime, a mask stimulus (randomly distributed white lines) was shown for 30 ms. This resulted in a stimulus onset asynchrony (SOA) of 60 ms between the onsets of the subliminal prime and the target arrow. The target (a central white arrow pointing either left or right) and two flankers (identical white arrows positioned above and below the target arrow) were presented simultaneously for 100 ms. Participants were instructed to focus on the central target arrow and ignore the flankers. They were then asked to indicate the direction of the central target arrow by pressing the left “Ctrl” button with their left index finger for a left‐pointing target arrow and the right “Ctrl” button with their right index finger for a right‐pointing target arrow. Each trial ended with the participant's first response or 2000 ms after target onset. If no response was recorded within this time frame, the trial was marked as a “miss.” The response–stimulus interval (RSI) between the participant's first response and the onset of the following trial varied randomly between 1000 and 1200 ms. Trials were categorized based on the direction of both the prime and target arrows. When the prime and target arrows pointed in the same direction, the trial was labeled as “compatible.” Conversely, if they pointed in opposite directions, the trial was labeled “incompatible.” Additionally, trials were classified as “congruent” when the flanker and target arrow pointed in the same direction and as “incongruent” when they pointed in opposite directions. This classification resulted in four conditions: compatible‐congruent, incompatible‐congruent, compatible‐incongruent, and incompatible‐incongruent.

Each participant completed 384 trials, equally distributed across four blocks (96 trials per block). Within each block, all possible combinations of prime compatibility, flanker congruency, and target direction were randomized and presented at equal frequencies. At the first appointment, participants completed a practice run of 16 trials to familiarize themselves with the task immediately after MPH/placebo administration (i.e., before the potential onset of full MPH drug effects). Participants were instructed to respond as quickly and precisely as possible. After each block (96 trials), participants could take a self‐timed break (i.e., to rest their eyes) and resume via button press. The experiment took on average approximately 15 min to complete.

### 2.6 Statistical Analysis: Behavioral and ^1^H‐MRS Data

The behavioral (i.e., response accuracy and correct response times) data were analyzed using repeated measures ANOVAs in SPSS version 29.0.0.0 (IBM Corp., Armonk, N.Y., USA). The analysis included “prime compatibility” (compatible vs. incompatible), “flanker congruency” (congruent vs. incongruent), and MPH/Placebo (MPH vs. placebo) as within‐subject factors, and “MPH dosage group” (low vs. medium) and order of drug administration (MPH on first vs. second appointment) as between‐subject factors. Significant main and interaction effects were examined with uncorrected post hoc *t*‐tests (García‐Pérez 2023). When a participant's behavioral accuracy/performance was below the chance level (< 50%) in at least one task condition, that case was marked as an outlier and no longer considered for all subsequent analyses. Descriptive data are given as mean ± standard error of the mean (SEM). Linear correlation analyses were conducted to investigate the relationship between MRS‐assessed neurotransmitter levels and task performance. Missing MRS metabolite values (due to movement in the scanner) were handled using standard pairwise exclusion via SPSS, ensuring that only complete data pairs were analyzed in each correlation. The neurotransmitter levels served as independent variables, and the behavioral measures as dependent variables. Fisher's r‐to‐z transformation was applied to facilitate a meaningful statistical comparison of the obtained correlation coefficients from two independent samples. This method tests the H0 that the two independent sample correlations are equal (Fisher 1921; Weaver and Wuensch 2013). The transformation was achieved by converting the correlation coefficients (r) into z‐scores, following a normal distribution. The z‐scores were then compared by calculating their difference, considering each standard error. Significant *p* values indicate that the two correlation coefficients are statistically different from each other. Whenever our a priori hypotheses were directed, post hoc tests were conducted one‐tailed to account for this. The achieved power of significant effects was determined post hoc for *α* = 5% using GPower software (Faul et al. 2007).

### 2.7 EEG Recording and Preprocessing

The participants' EEG signals were recorded from 60 Ag/AgCl electrodes in equidistant positions with a “QuickAmp” amplifier (Brain Products GmbH, Gilching, Germany) and the “BrainVision Recorder” software (Version 2.2), as in previous publications (e.g., Koyun, Talebi, et al. 2024; Koyun, Wendiggensen, et al. 2024). The ground electrode was positioned at the coordinates *θ* = 58, *φ* = 78, and the reference at Fpz (*θ* = 90, *φ* = 90). EEG signals were initially recorded at a sampling rate of 500 Hz, while electrode impedances were kept below 10 kΩ. First, EEG data were down‐sampled to 256 Hz, and flat channels were removed (i.e., channels that showed activity below 5 μV for more than 5 s). The remaining channels were then re‐referenced to an average reference. Subsequently, the PREP preprocessing pipeline (Bigdely‐Shamlo et al. 2015) was applied, which removes line noise (for data recorded in Europe: 50 Hz) using a multi‐taper algorithm. After removing contaminations by noisy/bad channels (using high and minimum variance criterion), a robust common average reference was applied. EOG artifacts were removed using the EOG Regression (Parra et al. 2005) subtraction method. Subsequently, the EEGLABs pop_eegfiltnew() pipeline was used to apply a high pass filter (cutoff frequency: 0.5 Hz) and low pass filter (cutoff frequency: 40 Hz); the filter order was estimated by default. Remaining artifactual source components in the data were detected by applying the Multiple Artifact Rejection Algorithm (MARA; Winkler et al. 2011), which automatizes independent component analyses (ICA). For the ICA, the data were temporarily high pass filtered with 1 Hz, but this option was not applied to the final pre‐processed data. In the final step, removed/missing channels were interpolated using a spherical method. After visual inspection of each data set, the EEG data were segmented into 4 s segments in a target‐locked manner (2 s before, and 2 s after the target) into the four trial conditions: compatible prime—congruent flanker, incompatible prime—congruent flanker, compatible prime—incongruent flanker, and incompatible prime—incongruent flanker. Only correct trials were considered for subsequent analyses.

To examine theta‐band (4–7 Hz) and alpha‐band (8–12 Hz) activity in these conditions, time‐frequency (TF) decomposition was performed using Morlet wavelets (with a width parameter of 5). The average power in the theta and alpha frequency bands was computed for each electrode and time point. Only significant behavioral effects, particularly those showing differences between the two MPH dosage groups, were further examined at the neurophysiological level (see behavioral results section). For this approach, cluster‐based permutation tests (CBPTs) were conducted to identify clusters of electrodes with significant differences between compatible and incompatible prime trials. The CBPTs were performed separately for each frequency band within 0–1000 ms after target arrow stimulus onset using 1000 Monte Carlo randomizations and a cluster‐alpha level of *p* = 0.025 (two‐tailed). Analyses were conducted separately for low and medium MPH dosage groups on the MPH appointment.

For later exploratory add‐on analyses (i.e., the correlation of behavioral and neurophysiological data), we further extracted ABA and TBA at electrode Cz (which was in the center of all identified clusters, compare Figure 6) in the entire time frame of 0–1000 ms, as well as the time frame of 10–600 ms (where the relevant condition difference was most evident, compare Figure 6) and correlated them to the obtained significant behavioral effects (for those details, please refer to the results section).

### 2.8 Source Estimation and Beamforming Analysis

First, dynamic imaging of coherent sources (DICS) beamforming (Gross et al. 2001) was applied to identify neuroanatomical sources of substantial differences between conditions of interest in the frequency domain. The source localization results were projected onto an equally spaced 0.5 cm grid created from the forward model template provided by the FieldTrip toolbox, based on the standard MNI (Montreal Neurological Institute) space. Power in the alpha and theta bands was extracted for the 1000 ms period following the presentation onset of the target stimulus. For both alpha and theta source power differences, corresponding contrasts were calculated and normalized on the total power of the two conditions as a ratio (Mückschel et al. 2016):

$$\text{Prime effect}\;\text{ratio}=\frac{{\text{Power}}_{\text{compatible prime}}-{\text{Power}}_{\text{incompatible prime}}}{{\text{Power}}_{\text{compatible prime}}+{\text{Power}}_{\text{incompatible prime}}}$$

Next, clusters of both frequency bands were identified by applying the density‐based spatial clustering of applications with noise (DBSCAN; Ester et al. 1996) algorithm as employed in MATLAB, comparable to previous studies from our group (e.g., Wendiggensen et al. 2022). The DICS beamforming results were restricted to negative ratios, indicating that theta and alpha power were higher in incompatible than in compatible trials. The negative top 1% of the power distribution in the prime effect ratio within labeled regions on the automated anatomical labeling atlas (Tzourio‐Mazoyer et al. 2002) were submitted to the DBSCAN, restricting the analysis to the voxels with the largest negative differences. An epsilon of 1.5 the edge length of each voxel was used to detect neighboring voxels.

**FIGURE 3:**
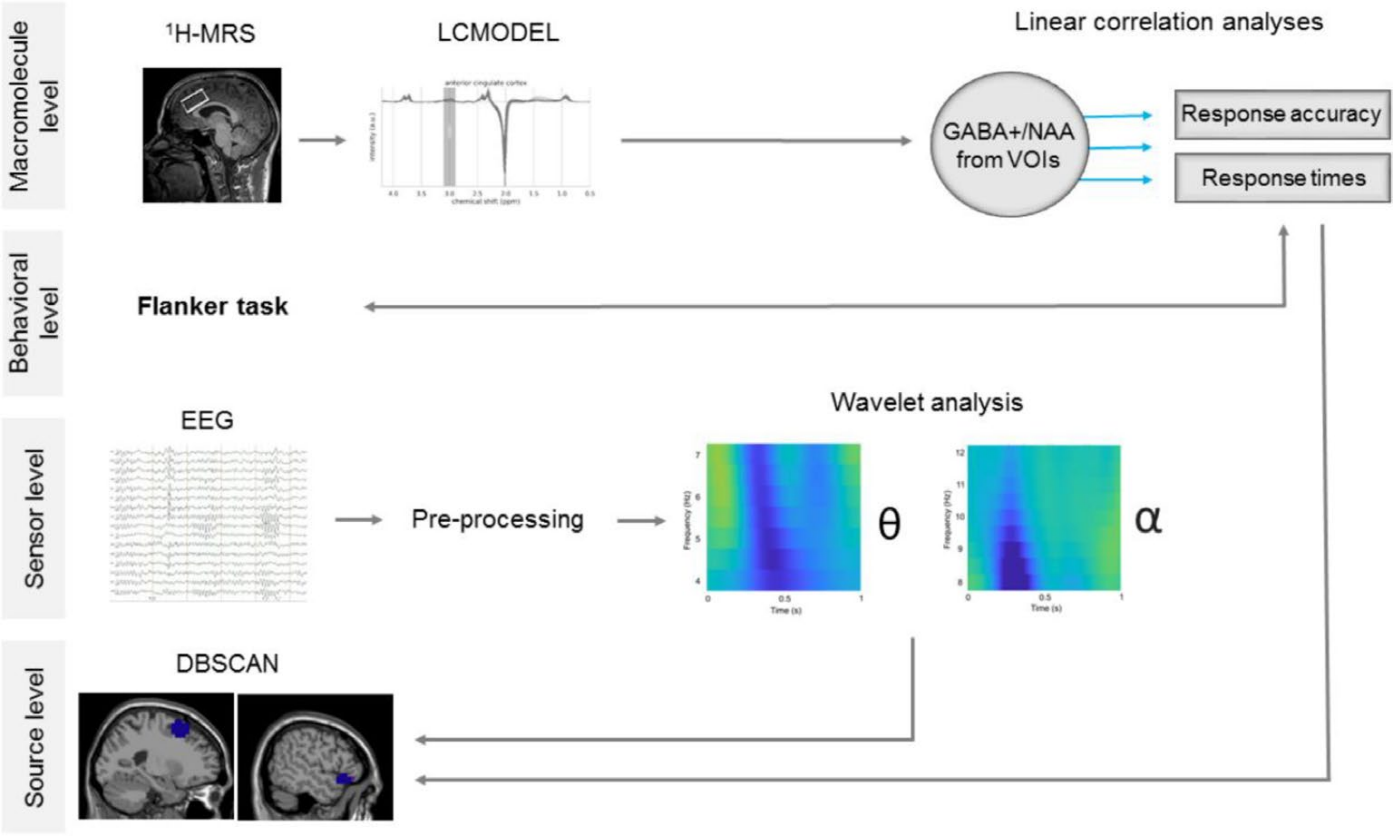
Schematic overview of applied methods and analyses. Illustrated is a visual summary of all methods and analyses applied from macromolecule level to behavioral level, over sensor to source level. It also depicts how we linked features from the macromolecule level to behavioral outcome measures by applying linear correlation analyses.

A schematic overview of all applied methods and analyses is provided in Figure 3.

## 3 Results

### 3.1 Behavioral Data

Given the extensive experimental design, the sections below are limited to the relevant statistically significant effects (i.e., general task effects, which are defined by the interaction of prime * flanker and effects of interest including the factor of pharmacological group, which distinguishes between low vs. medium MPH dose). The full outcome of the ANOVAs as well as descriptive data for each combination of factors in each group is provided in the supplement.

#### 3.1.1 Task Effects: Proof of Principle

The usual effects of prime compatibility and flanker congruency were replicated (e.g., Bensmann et al. 2018; Stock et al. 2016), with higher accuracy and lower hit response times observed in congruent/compatible trials than in incongruent/incompatible ones.

We further found increased conflict effects if the other conflict was also present, which is the typical finding for this task (see e.g., Stock et al. 2016). Specifically, there was an interaction of prime compatibility × flanker congruency for accuracy (*F*_(1,72)_ = 55.042; *p* < 0.001; $\eta_{p}^{2}$ = 0.433; achieved power > 99%) and for response times (*F*_(1,72)_ = 14.923; *p* < 0.001; $\eta_{p}^{2}$ = 0.172; achieved power > 99%). Post hoc *t*‐tests revealed significant differences for all possible contrasts (all *p* ≤ 0.001). In line with previous results, the prime compatibility effect (i.e., compatible—incompatible for accuracy and vice versa for response times) was larger in incongruent flankers (accuracy: 10.263% ± 1.167; response times: 45.497 ms ± 3.102) than in congruent flankers (accuracy: 5.632% ± 0.771; response times: 40.194 ms ± 2.661) [accuracy: *t*_(1,75)_ = −7.390; *p* < 0.001; response times: *t*_(1,75)_ = 3.943; *p* < 0.001]. Additionally, the flanker congruency effect (i.e., congruent—incongruent for accuracy and vice versa for response times) was larger in incompatible primes (accuracy: 7.424% ± 0.788; response times: 23.540 ms ± 1.262) than in compatible primes (accuracy: 2.793% ± 0.416; response times: 18.238 ms ± 1.473) [accuracy: *t*_(1,75)_ = −7.390; *p* < 0.001; response times: *t*_(1,75)_ = 3.943; *p* < 0.001].

Furthermore, there was an interaction of prime compatibility × flanker congruency × MPH dosage group for accuracy (*F*_(1,72)_ = 4.024; *p* = 0.049; $\eta_{p}^{2}$ = 0.053; achieved power > 99%). Post hoc *t*‐tests comparing the magnitude of the prime and flanker effect in the presence of the respective other conflict were conducted separately for each pharmacological group. For low and medium MPH dosage groups, the prime and flanker effects were significantly larger in the presence of the respective other conflict. Specifically, for both groups larger prime effects were shown when the flanker was incongruent (low dosage group: 11.930% ± 1.768, medium dosage group: 8.596% ± 1.499) than congruent (low dosage group: 6.073% ± 1.292, medium dosage group: 5.190% ± 0.855). Furthermore, the flanker effect in both pharmacological groups was larger in trials in which the prime was incompatible (low dosage group: 8.307% ± 1.017, medium dosage group: 6.542% ± 1.200) as compared to compatible prime trials (low dosage group: 2.450% ± 0.507, medium dosage group: 3.136% ± 0.662).

#### 3.1.2 Further MPH Dosage Group Effects: Relevant MPH Dose Effects

**FIGURE 4:**
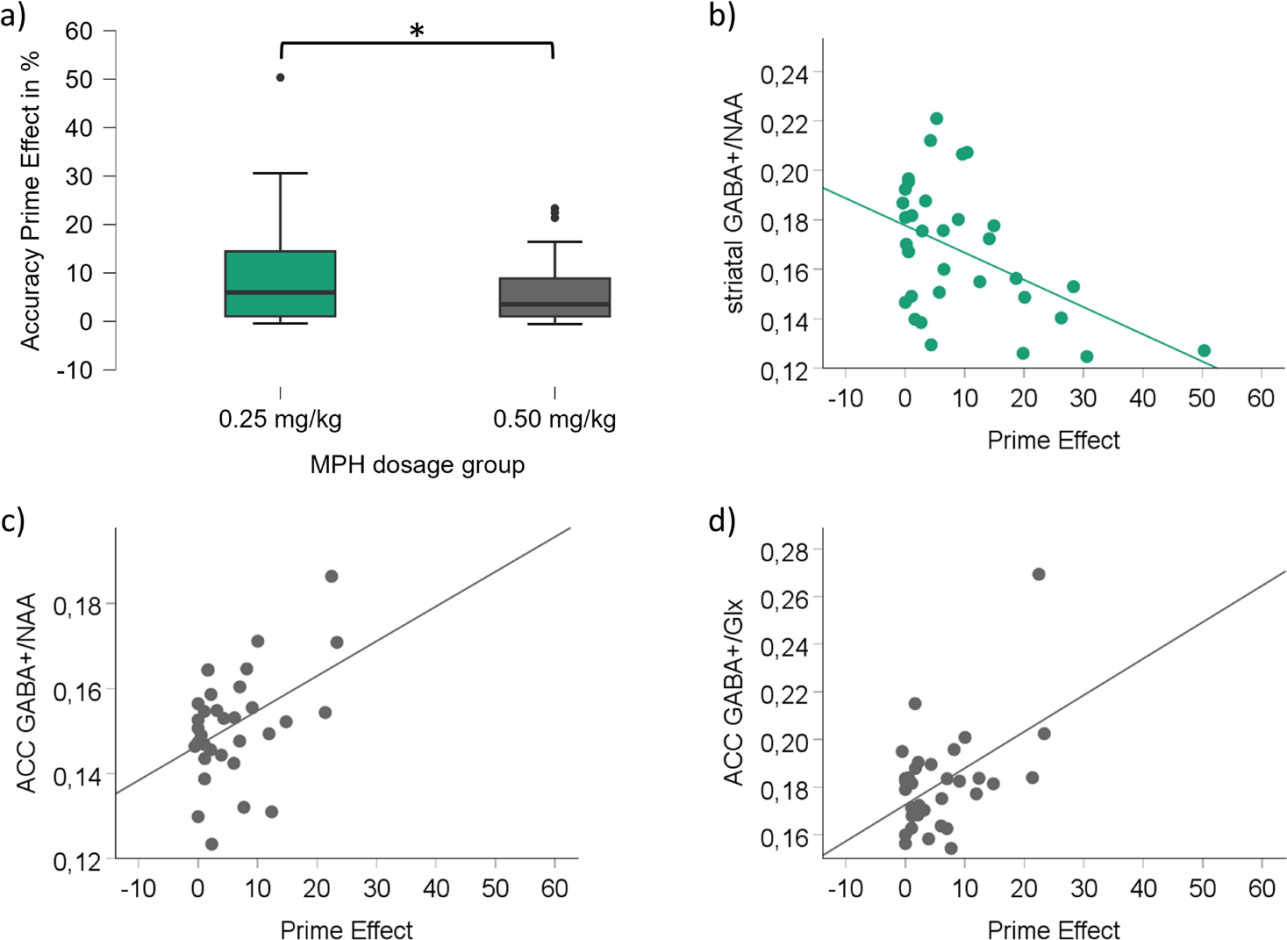
Behavioral accuracy results and MRS scatterplots. (a) Illustrated is the accuracy prime effect (compatible minus incompatible trials) for low (green) and medium (grey) MPH dosage groups on the MPH appointment. The asterisk indicates significant differences at *p* < 0.05, and the error bars represent the 95% confidence intervals. The scatterplots in (b), (c), and (d) illustrate the relationship between ^1^H‐MRS measures and the accuracy prime effect on the MPH appointment. (b) For the low MPH dosage group (c) and (d) for the medium dosage group. The *x*‐axis denotes the prime in % on the MPH appointment. The *y*‐axis denotes the respective amino acid neurotransmitter levels. Only significant correlations are depicted.

The repeated measures ANOVA further revealed an interaction of MPH/placebo × prime compatibility × MPH dosage group for accuracy (*F*_(1,72)_ = 4.077; *p* = 0.047; $\eta_{p}^{2}$ = 0.054; achieved power > 99%). Post hoc *t*‐tests for the MPH appointment showed significantly larger prime effects (compatible minus incompatible) for the group that received low MPH doses (9.595% ± 1.788) as compared to individuals who received medium doses of MPH (5.915% ± 1.080) [*t*_(1,60.841)_ = 1.761; one‐sided *p* = 0.042]. On the placebo appointment, no such difference between the MPH dosage groups was evident (two‐sided *p* = 0.793). The accuracy results are illustrated in Figure 4a.

**FIGURE 5:**
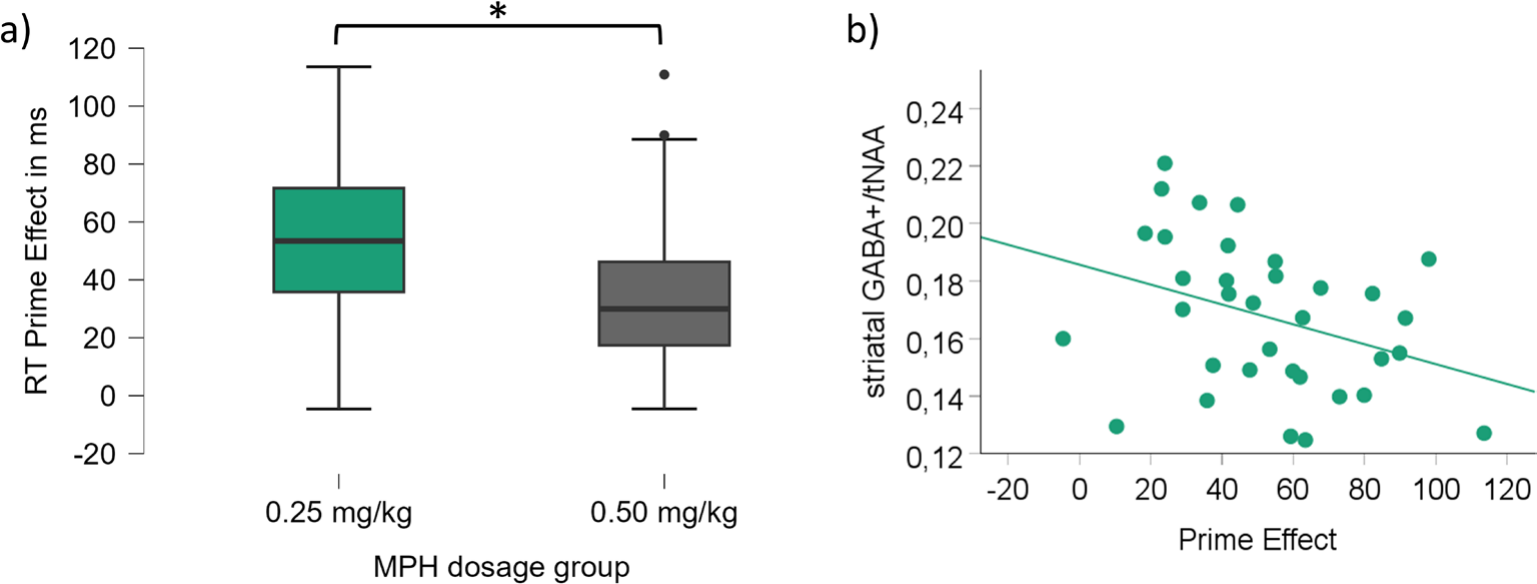
Response times results and MRS scatterplots. (a) Illustrated is the response times prime effect (incompatible minus compatible trials) for low (green) and medium (grey) MPH dosage groups on the MPH appointment. The asterisk indicates significant differences at *p* < 0.05, and the error bars represent the 95% confidence intervals. (b) Illustrated is the relationship between the GABA+/tNAA and the response times prime effect for the low MPH dosage group on the MPH appointment. The *x*‐axis denotes the response times' prime effect in ms on the MPH appointment. The *y*‐axis denotes the GABA+/tNAA levels. Only significant correlations are depicted.

Mirroring the accuracy results, there was an interaction of MPH/placebo × prime compatibility × MPH dosage group for response times (*F*_(1,72)_ = 7.630; *p* = 0.007; $\eta_{p}^{2}$ = 0.096; achieved power > 99%). Subsequent post hoc t‐tests for the MPH appointment indicated significantly larger prime effects (incompatible minus compatible) in the low MPH dosage group (54.021 ms ± 4.399) than in the medium MPH dosage group (33.665 ms ± 4.191) [*t*_(1,74)_ = 3.350; one‐sided *p* < 0.001]. On the placebo appointment, no difference between the MPH dosage groups was evident (two‐sided *p* = 0.315). The response times results are illustrated in Figure 5a.

Given that the same interaction effect was found for both accuracy and response times, we conducted additional post hoc t‐tests to investigate a potential speed‐accuracy tradeoff. Doing so revealed that while the prime effect (i.e., the difference measure) significantly differed between the low and medium dose groups in the MPH appointment, the compatible (low MPH: 98.171% ± 0.404 and 361.857 ms ± 6.206; medium MPH: 97.386% ± 0.573 and 373.902 ms ± 6.548) and incompatible (low MPH: 88.576% ± 1.879 and 415.878 ms ± 5.357; medium MPH: 91.470% ± 1.441 and 407.567 ms ± 5.445) prime conditions did not significantly differ between MPH dosage groups for either accuracy or response times (all *p* ≥ 0.186). This demonstrates that the MPH‐associated prime effect differences reported could not be traced back to differences in only one task condition and were also not due to an underlying speed‐accuracy‐tradeoff.

### 3.2 ^1^H‐MRS‐Assessed Transmitter Levels as a Modulator of the Subconscious Conflict Effects After MPH Administration

Given the prior behavioral finding of smaller prime effects after medium (as compared to small) MPH doses, we set out to investigate whether and how this effect was linked to the baseline levels of GABA+ and Glx: Separate linear correlation analyses were conducted for each dosage group to assess whether the ^1^H‐MRS‐measured transmitter levels in each of the three VOIs correlated with the prime effect (accuracy and response times) during the MPH session. The findings show clear differences between the low and medium dosage groups (see Tables 1 and 2): For the low MPH dose group, only striatal ^1^H‐MRS measures (i.e., GABA+/tNAA) correlated with the prime effect (both accuracy and response times), whereas for the medium dose group, only ACC ^1^H‐MRS measures (i.e., GABA+/tNAA and GABA+/Glx) significantly correlated with the prime effect (accuracy only). All significant correlations are illustrated in Figures 4 and 5.

**TABLE 1:** Correlation of 1H‐MRS measures and the MPH‐modulated prime effect: Accuracy.

VOI	Transmitter	Low MPH dose group		Medium MPH dose group		Fisher's *r*‐to‐*z* transformation	
		*r*	*p*	*r*	*p*	*z*‐score	*p*
Striatum	GABA+/tNAA	−0.479**	0.004	−0.017	0.923	−1.987	0.023*
	Glx/tNAA	−0.247	0.159	−0.200	0.257	−0.195	0.423
	GABA+/Glx	−0.329	0.058	0.068	0.701	−1.613	0.053
ACC	GABA+/tNAA	−0.019	0.917	0.430*	0.013	−1.870	0.031*
	Glx/tNAA	0.125	0.482	−0.214	0.231	1.339	0.090
	GABA+/Glx	−0.066	0.713	0.485**	0.004	−2.326	0.010*
SMA	GABA+/tNAA	0.016	0.931	0.010	0.959	0.023	0.491
	Glx/tNAA	0.211	0.246	0.094	0.615	0.453	0.325
	GABA+/Glx	−0.102	0.580	0.058	0.760	−0.600	0.274

*Note:* The four middle columns report the Pearson correlation of each VOI and transmitter with the accuracy MPH prime effect in the low and medium dose groups, respectively. The *r*‐to‐*z* transformation flags significant MPH dose group differences in the strength of these linear relationships. *p* values of the correlation analyses are two‐sided, while the *p* values of Fisher's *r*‐to‐*z* transformation are one‐sided. Significant effects of *p* ≤ 0.05 are marked with one asterisk, while significant effects of *p* ≤ 0.01 are marked with two asterisks. The achieved power for the significant correlations found in each group was > 99%. The achieved power for the significant *r*‐to‐*z* results was 63% for striatal GABA+/tNAA, 59% for ACC GABA+/tNAA, and 75% for ACC GABA+/Glx.

Abbreviations: ACC = anterior cingulate cortex; SMA = supplementary motor area; VOI = volume of interest.

**TABLE 2:** Correlation of 1H‐MRS measures and the MPH‐modulated prime effect: Response times.

VOI	Transmitter	Low MPH dose group		Medium MPH dose group		Fisher's *r*‐to‐*z* transformation	
		*r*	*p*	*r*	*p*	*z*‐score	*p*
Striatum	GABA+/tNAA	−0.352*	0.041	0.026	0.885	−1.550	0.061
	Glx/tNAA	−0.219	0.214	−0.054	0.761	−0.664	0.253
	GABA+/Glx	−0.243	0.167	0.039	0.829	−1.130	0.129
ACC	GABA+/tNAA	−0.143	0.421	0.188	0.296	−1.305	0.096
	Glx/tNAA	0.130	0.464	−0.060	0.741	0.745	0.228
	GABA+/Glx	−0.222	0.207	0.216	0.228	−1.115	0.132
SMA	GABA+/tNAA	−0.109	0.552	0.046	0.806	−0.587	0.279
	Glx/tNAA	−0.072	0.693	0.002	0.991	−0.280	0.390
	GABA+/Glx	−0.065	0.725	−0.076	0.689	−0.041	0.484

*Note:* The four middle columns report the Pearson correlation of each VOI and transmitter with the response time MPH prime effect in the low and medium dose groups, respectively. The *r*‐to‐*z* transformation flags significant MPH dose group differences in the strength of these linear relationships. *p* values of the correlation analyses are two‐sided, while the *p* values of the Fisher's *r*‐to‐*z* transformation are one‐sided. Significant effects of *p* ≤ 0.05 are marked with one asterisk, while significant effects of *p* ≤ 0.01 are marked with two asterisks. The achieved power for the significant correlations found in each group was 99%.

Abbreviations: ACC = anterior cingulate cortex; SMA = supplementary motor area; VOI = volume of interest.

To assess whether the strength of these linear relationships significantly differed between the two MPH dosage groups, we additionally ran Fisher's *r*‐to‐*z* transformation. For the accuracy prime effect, this revealed that in each case where one of the dose groups had a significant correlation (but the other did not), the group with the significant correlation also had a significantly stronger linear relationship (as compared to the group without the significant correlation) (see Table 1). This pattern was, however, absent from the response time data (see Table 2).

### 3.3 Neurophysiological Results

Given the prior behavioral finding of smaller prime effects after medium (as compared to small) MPH doses, the investigation of neurophysiological differences between the MPH dosage groups was restricted to the magnitude of the prime effect on the MPH appointment.

**FIGURE 6:**
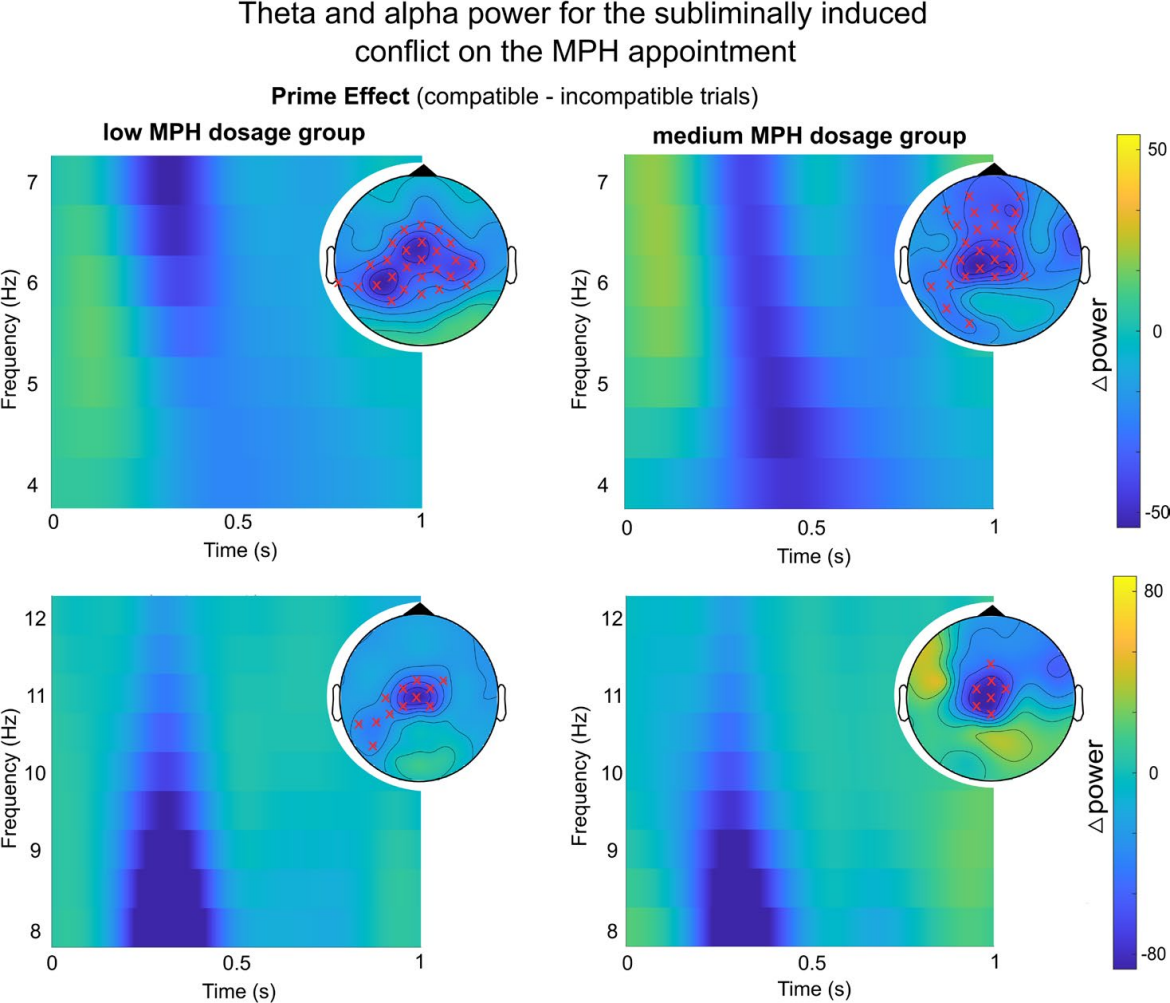
Time‐frequency (TF) analysis and cluster‐based permutation testing (CBPT) results. The TF plots display average power differences (0–1000 ms post‐target arrow onset) for the prime effect (compatible MINUS incompatible trials) on the MPH appointment. Data for the theta band is depicted in the top row and alpha band data in the bottom row. Topographic plots highlight significant electrodes identified in the CBPTs, marked with red “*x*”s to indicate negative differences. These plots show average power in the significant timeframe (100–600 ms) for both low (left row) and medium (right row) MPH dosage groups.

In the interval of 0–1000 ms relative to target onset, cluster‐based permutation tests (CBPTs) were separately conducted for alpha and theta band activity (ABA and TBA, respectively), and separately for low and medium MPH dosage groups. The results revealed significant negative differences (i.e., more activity in incompatible than in compatible trials) in both ABA and TBA. The significant electrode locations of the cluster‐based permutation tests are visualized in Figure 6, accompanied by the time‐frequency representation (TFRs) of the power difference between prime conditions (congruent—incongruent) on the MPH appointment.

**FIGURE 7:**
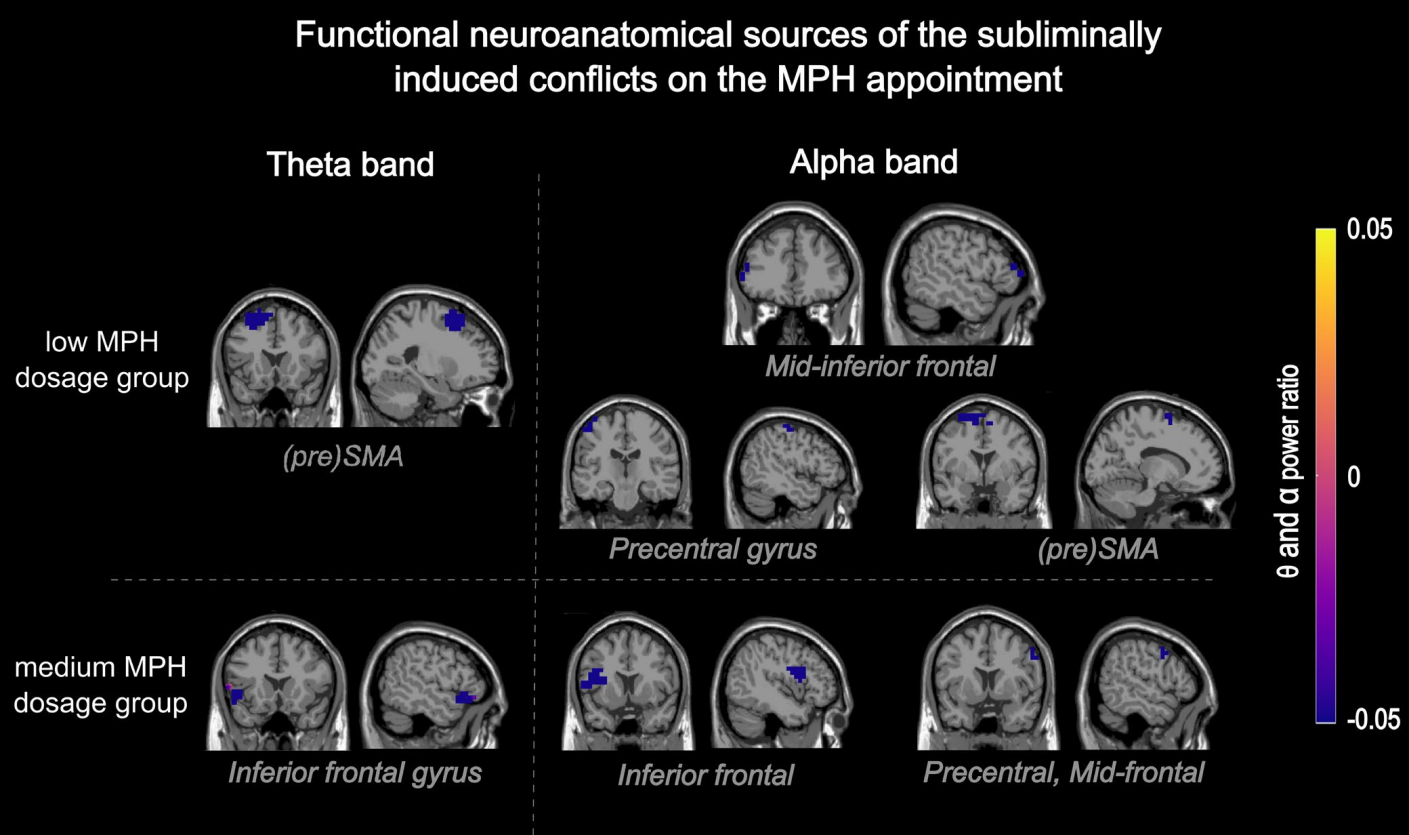
Neuroanatomical clusters associated with the subliminally induced response conflict on the MPH appointment. Depicted are the source‐localized activity in theta and alpha bands as identified by the DBSCAN algorithm. The color of the cluster represents the power ratio (as detailed in the methods section) of the prime effect (compatible minus incompatible). On the left are clusters identified in the theta band, and on the right, clusters identified in the alpha band.

On the source level, the DBSCAN algorithm applied on the prime effect ratio identified several negative clusters in both alpha and theta bands for both low and medium MPH dosage groups. An overview of all resulting clusters is provided in Figure 7.

As a final exploratory analysis, we further correlated the ABA and TBA prime effects (at electrode Cz, in the time windows of 0–1000 ms and 100–600 ms) with the behavioral prime effects on the MPH appointment. For the accuracy prime effect, no significant correlations were found with ABA or TBA prime effects for either MPH group (see Table 3). For the response time prime effect, both MPH dosage groups showed a significant correlation with the TBA prime effect, and the medium MPH group additionally showed a significant correlation with the ABA prime effect in the shorter time window (see Table 4).

**TABLE 3:** Correlation of neurophysiological measures and the MPH‐modulated prime effect: Accuracy.

Frequency band	Time window	Low MPH dose group		Medium MPH dose group		Fisher's *r*‐to‐*z* transformation	
		*r*	*p*	*r*	*p*	*z*‐score	*p*
TBA	0–1000 ms	0.021	0.889	−0.024	0.887	0.188	0.425
	100–600 ms	0.044	0.794	−0.080	0.635	0.520	0.302
ABA	0–1000 ms	−0.101	0.547	−0.035	0.834	−0.278	0.391
	100–600 ms	−0.072	0.666	−0.180	0.280	0.460	0.323

*Note:* The four middle columns report the Pearson correlation of the mean frequency band activity prime effect (calculated as compatible minus incompatible) with the accuracy MPH prime effect (calculated as compatible minus incompatible) in the low and medium dose groups, respectively. The *r*‐to‐*z* transformation flags significant MPH dose group differences in the strength of these linear relationships. *p* values of the correlation analyses are two‐sided, while the *p* values of the Fisher's *r*‐to‐*z* transformation are one‐sided. Significant effects of *p* ≤ 0.05 are marked with one asterisk, while significant effects of *p* ≤ 0.01 are marked with two asterisks.

Abbreviations: ABA = alpha band activity; TBA = theta band activity.

**TABLE 4:** Correlation of neurophysiological measures and the MPH‐modulated prime effect: Response times.

Frequency band	Time window	Low MPH dose group		Medium MPH dose group		Fisher's *r*‐to‐*z* transformation	
		*r*	*p*	*r*	*p*	*z*‐score	*p*
TBA	0–1000 ms	−0.477**	0.002	−0.675**	< 0.001	1.258	0.104
	100–600 ms	−0.477**	0.002	−0.682**	< 0.001	1.313	0.095
ABA	0–1000 ms	−0.004	0.980	−0.252	0.127	1.060	0.144
	100–600 ms	−0.201	0.227	−0.389*	0.013	0.865	0.193

*Note:* The four middle columns report the Pearson correlation of the mean frequency band activity prime effect (calculated as compatible minus incompatible) with the response time MPH prime effect (calculated as incompatible minus compatible) in the low and medium dose groups, respectively. The *r*‐to‐*z* transformation flags significant MPH dose group differences in the strength of these linear relationships. *p* values of the correlation analyses are two‐sided, while the *p* values of the Fisher's *r*‐to‐*z* transformation are one‐sided. Significant effects of *p* ≤ 0.05 are marked with one asterisk, while significant effects of *p* ≤ 0.01 are marked with two asterisks. The achieved power for the significant correlations found in each group for TBA was 99%, while the achieved power for the significant correlation in ABA was 95%.

Abbreviations: ABA = alpha band activity; TBA = theta band activity.

## 4 Discussion

In this study, we investigated how an experimental increase in catecholaminergic signaling modulates the relationship between baseline amino acid transmitter levels (in the ACC, striatum, and SMA) and interference control, as reflected by behavioral performance and TBA/ABA. Most importantly, the results showed that while the size of the prime effect did not differ between groups during the placebo appointment (as it should), medium doses of MPH seemed to produce a significantly smaller prime effect than low MPH doses. Our findings further show that baseline concentrations of GABA+ and GABA+/Glx in cognitive control‐relevant regions modulate how MPH affects response control performance: under moderate doses of MPH, lower GABA+ and GABA+/Glx levels in the ACC are associated with more efficient subliminal interference control (i.e., a relatively smaller prime effect). Conversely, higher striatal GABA+ concentrations were associated with more efficient interference control (i.e., a relatively smaller prime effect) under low doses of MPH. Importantly, those findings do not only support our main hypotheses of improved interference control in the case of mild to moderate enhancement of catecholaminergic signaling as well as in the case of greater striatal inhibition and greater ACC excitation, they also highlight that whether and how amino acid transmitter levels in cognitive control‐relevant regions modulate (subliminal) conflicts depends on the degree of catecholaminergic signaling, as the relevant predictive region changed from striatal to cingulate with increasing catecholaminergic stimulation.

Interestingly, behavioral dose‐related differences appeared only in response to subliminal conflicts, highlighting the specific modulation of subconscious conflict processing efficiency by medium MPH doses. This suggests that MPH‐induced catecholaminergic effects on cognitive control are particularly strong in subconsciously processed conflicts, likely due to enhanced integration and filtering of subliminal information (Ivanov et al. 2014). Further, the fact that the medium MPH group demonstrated a smaller prime effect than the low MPH group suggests that on average, we did not over‐stimulate our participants past the point of optimal performance on the inverted U‐shaped curve (Arnsten 2011; Bar‐Gad et al. 2003; Bensmann et al. 2020; Bensmann, Zink, Arning, et al. 2019; Petzold et al. 2019; Robbins and Arnsten 2009).

The established associations between baseline amino acid transmitter levels and interference effects were specific to the degree of catecholaminergic stimulation and cortical area. In the low‐dose group, greater striatal inhibition (reflected by higher GABA+ levels) was associated with smaller subliminal interference effects in accuracy and response times. This suggests that, at slightly elevated catecholamine signaling, striatal GABA+ is crucial for modulating/improving interference control, consistent with previous findings that striatal GABA improves conflict monitoring, information filtering, and response control (Haag et al. 2015; Yildiz et al. 2014). Striatal GABA supports the “winner‐takes‐all” (WTA) network, where inhibitory interconnections suppress competing actions, favoring one outcome (Plenz 2003). MPH‐induced DA increases likely enhance GABA release from striatal interneurons (Freese et al. 2012; Tritsch and Sabatini 2012), reinforcing this WTA network and improving attentional gating (Yildiz et al. 2014).

Conversely, lower inhibition/greater excitation (i.e., lower GABA+ and GABA+/Glx levels) in the ACC were associated with more efficient interference control at medium MPH doses, reflecting the ACC's well‐established role in conflict monitoring and resolution (Botvinick 2007). This dose‐dependent shift from striatal to ACC neurotransmitter dominance suggests a reorganization of cognitive control mechanisms, with the ACC playing a more central role in resolving subliminal interference, highlighting a potential mechanism by which MPH enhances the ACC's contribution at medium doses. Given relatively high GABA levels in the ACC (Whissell et al. 2015), the GABAergic and glutamatergic systems are critical for distinguishing between specific inputs and impulsivity control, facilitating effective conflict and response control (Adams et al. 2017; Fujihara et al. 2015; Takacs et al. 2021). The greater reliance on ACC GABA+/Glx activity at medium doses likely explains the enhanced conflict resolution and reduced interference effects seen at medium doses, indicating the ACC's broader role in coordinating high‐level cognitive control, conflict resolution, and adaptive goal‐directed behavior (Adams et al. 2017; Bari and Robbins 2013). Notably, GABA+ and Glx levels in the SMA were not significant predictors of interference control in either dosage group, consistent with the hypothesis that the SMA, though involved in motor response control and execution (Coull et al. 2016; Nachev et al. 2005), plays a less central role in MPH‐modulated processing of subliminal response conflicts than the ACC and striatum.

The shift in the locus of neurochemical importance suggests that catecholaminergic signaling fine‐tunes interference control through dose‐ and region‐specific pathways, thereby also offering new insights into how MPH alters neural oscillatory dynamics during cognitive tasks. Consistent with previous research on interference control, changes in oscillatory activity, such as increased TBA in high‐demand conflict tasks, demonstrate the interplay between neurotransmitter regulation and network‐level oscillations (Koyun, Talebi, et al. 2024). Regardless of MPH dosage, an increased need for interference control was reflected in higher TBA and ABA in incompatible compared to compatible primes (Berchou et al. 1986; Cavanagh and Frank 2014; Koyun, Talebi, et al. 2024) and a more distributed cortical network associated with interference control in ABA. In the alpha band, subliminal conflict processing was associated with mid‐superior‐frontal and inferior‐frontal regions in both dosage groups. Alpha band oscillations are associated with inhibiting task‐irrelevant information (Foxe and Snyder 2011) by suppressing irrelevant neural activity before stimulus presentation, potentially resolving subliminal response conflicts before reaching conscious awareness (Foxe and Snyder 2011; Jensen and Mazaheri 2010; Klimesch et al. 2007). It is hence in line with our hypotheses that ABA was higher in incompatible than in compatible trials. The observed ABA clusters indicate that both the primary motor cortex (M1) and prefrontal regions, including the IFG and (pre)SMA, are integral to this process. ABA modulations in M1 likely reflect the suppression of task‐irrelevant or distracting stimulus information challenging existing action plans (Calderon et al. 2018; Pape and Siegel 2016; Rhodes et al. 2018). Simultaneously, the IFG and (pre)SMA play complementary roles, managing conflicts between subliminal primes and planned responses (Moss et al. 2005; Sadaghiani and Kleinschmidt 2016; Swick et al. 2008), indicating ABA's role in prioritizing relevant information while inhibiting irrelevant inputs. The (pre)SMA, particularly in the low MPH dosage group, appears to enhance both attentional and cognitive control (Deiber et al. 2012; Sadaghiani and Kleinschmidt 2016), facilitating conflict resolution. Our results suggest that ABA modulations across mid‐superior‐frontal and inferior‐frontal regions are critical for interference control, mainly when it involves subliminal conflicts. MPH may amplify the alpha‐band's role in gating sensory information and resolving subliminal conflicts by sharpening the motor plan and reducing errors from conflicting subliminal information (Beste et al. 2023).

On the MPH appointment, a larger TBA prime effect was stably associated with larger behavioral conflict effects (in the response time measure) in both groups, which emphasizes the well‐known functional link between TBA and interference control (Beste et al. 2023; Cavanagh and Frank 2014; Klimesch 2012). Crucial differences between the MPH dosage groups emerged in the neuroanatomical sources of TBA, indicating catecholamine‐dependent neural dynamics and reflecting the role of TBA in stimulus and response selection during cognitive control (Mückschel, Dippel, and Beste 2017): In the low‐dosage group, the subliminal conflict was reflected in TBA modulations in the (pre)SMA, where TBA increases under high cognitive control demands, particularly with conflicting information (Cavanagh and Frank 2014; Koyun, Wendiggensen, et al. 2024; Koyun, Talebi, et al. 2024). It is hence in line with our hypotheses that TBA was higher in incompatible than in compatible trials. This aligns with TBA in the (pre)SMA being sensitive to MPH‐induced allocation of attentional resources and response control in demanding conditions (Koyun, Talebi, et al. 2024; Pauls et al. 2012; Rubia et al. 2011). In the medium dosage group, the subliminal conflict was associated with TBA in the left IFG, emphasizing its role during conflict detection, resolution (Moss et al. 2005; Zhang et al. 2004), and modulating neural circuits under varying cognitive loads (Koyun, Wendiggensen, et al. 2024). The MPH dose‐dependent neuroanatomical specificity in TBA sources may elucidate the behavioral outcomes, where low dosages promote more focused and stable cognitive control. In contrast, medium dosages enable a more flexible and broader distribution of cognitive resources to address complex tasks (Diveica et al. 2023). TBA may thus be a critical marker for understanding the complex effects of catecholaminergic signaling on cognitive control mechanisms.

Lastly, it needs to be mentioned that we did not systematically assess the efficiency of the blinding procedure. We can therefore not exclude the possibility that different proportions of participants in each dosage group correctly guessed on which appointments the placebo and MPH were administered.

## 5 Conclusion

In summary, our study reveals how catecholaminergic signaling modulates amino acid‐driven subliminal conflict processing in a dose‐dependent manner in control‐associated brain regions and oscillatory mechanisms. At low levels of catecholaminergic stimulation, striatal GABA+ enhances conflict monitoring and attentional gating. In contrast, medium catecholaminergic stimulation shifts the focus of interference control to the ACC, where GABA+/Glx signaling is used for conflict resolution. These dose‐dependent mechanisms align with oscillatory dynamics. TBA adapts to cognitive demands in the (pre)SMA and IFG depending on MPH dosage (Cavanagh and Frank 2014; Koyun, Talebi, et al. 2024; Koyun, Wendiggensen, et al. 2024). Conversely, ABA networks remain crucial across doses, gating and resolving subliminal conflicts (Foxe and Snyder 2011; Jensen and Mazaheri 2010), thereby highlighting the fact that catecholaminergic signaling fine‐tunes neural circuits for efficient cognitive control in a frequency band‐specific manner. The results suggest that baseline neurotransmitter levels significantly affect responses to pharmacological interventions like MPH, suggesting that different brain regions might drive the same cognitive processes based on inter‐individual neurochemical states.

## Author Contributions

Conceptualization: A.‐K.S., C.B. Data curation: A.H.K. Formal analysis: A.H.K., A.‐K.S., A.W., P.K. Funding acquisition: A.‐K.S., C.B., V.R. Investigation: A.H.K., A.W. Project administration: A.‐K.S., C.B. Software: A.W., P.K. Supervision: A.‐K.S., C.B., V.R. Validation: A.H.K. Visualization: A.H.K., A.‐K.S., A.W. Writing – original draft: A.H.K., A.W., A.‐K.S. Writing – review and editing: All authors.

## Ethics Statement

The study was approved by the ethics committee of TU Dresden (EK 420092015) and conducted following the Declaration of Helsinki (Rickham 1964) and its later amendments.

## Consent

All participants provided written consent and were financially reimbursed for their participation.

## Conflicts of Interest

The authors declare no conflicts of interest.

## Supporting information


**Data S1:** Supporting Information.

## Data Availability

The data that support the findings of this study are available on request from the corresponding author. The data are not publicly available due to privacy or ethical restrictions.

## References

[hbm70385-bib-0001] Adams, N. E. , J. S. Sherfey , N. J. Kopell , M. A. Whittington , and F. E. N. LeBeau . 2017. “Hetereogeneity in Neuronal Intrinsic Properties: A Possible Mechanism for Hub‐Like Properties of the Rat Anterior Cingulate Cortex During Network Activity.” eNeuro 4: ENEURO.0313‐16.2017. 10.1523/ENEURO.0313-16.2017. PMC5340498 28275720

[hbm70385-bib-0003] Arnsten, A. F. T. 2011. “Catecholamine Influences on Dorsolateral Prefrontal Cortical Networks.” Biological Psychiatry 69: e89–e99. 10.1016/j.biopsych.2011.01.027. 21489408 PMC3145207

[hbm70385-bib-0004] Baeshen, A. , P. O. Wyss , A. Henning , et al. 2020. “Test‐Retest Reliability of the Brain Metabolites GABA and Glx With JPRESS, PRESS, and MEGA‐PRESS MRS Sequences in Vivo at 3T.” Journal of Magnetic Resonance Imaging 51: 1181–1191. 10.1002/jmri.26921. 31667944

[hbm70385-bib-0005] Bar‐Gad, I. , G. Morris , and H. Bergman . 2003. “Information Processing, Dimensionality Reduction and Reinforcement Learning in the Basal Ganglia.” Progress in Neurobiology 71: 439–473. 10.1016/j.pneurobio.2003.12.001. 15013228

[hbm70385-bib-0006] Bari, A. , and T. W. Robbins . 2013. “Inhibition and Impulsivity: Behavioral and Neural Basis of Response Control.” Progress in Neurobiology 108: 44–79. 10.1016/j.pneurobio.2013.06.005. 23856628

[hbm70385-bib-0007] Bensmann, W. , V. Roessner , A.‐K. Stock , and C. Beste . 2018. “Catecholaminergic Modulation of Conflict Control Depends on the Source of Conflicts.” International Journal of Neuropsychopharmacology 21: 901–909. 10.1093/ijnp/pyy063. 30016467 PMC6165959

[hbm70385-bib-0008] Bensmann, W. , N. Zink , L. Arning , C. Beste , and A.‐K. Stock . 2019. “The Presynaptic Regulation of Dopamine and Norepinephrine Synthesis Has Dissociable Effects on Different Kinds of Cognitive Conflicts.” Molecular Neurobiology 56: 8087–8100. 10.1007/s12035-019-01664-z. 31183808

[hbm70385-bib-0009] Bensmann, W. , N. Zink , L. Arning , C. Beste , and A.‐K. Stock . 2020. “Dopamine D1, but Not D2, Signaling Protects Mental Representations From Distracting Bottom‐Up Influences.” NeuroImage 204: 116243. 10.1016/j.neuroimage.2019.116243. 31610297

[hbm70385-bib-0010] Bensmann, W. , N. Zink , V. Roessner , A.‐K. Stock , and C. Beste . 2019. “Catecholaminergic Effects on Inhibitory Control Depend on the Interplay of Prior Task Experience and Working Memory Demands.” Journal of Psychopharmacology 33: 678–687. 10.1177/0269881119827815. 30816793

[hbm70385-bib-0011] Berchou, R. , S. Chayasirisobhon , V. Green , and K. Mason . 1986. “The Pharmacodynamic Properties of Lorazepam and Methylphenidate Drugs on Event‐Related Potentials and Power Spectral Analysis in Normal Subjects.” Clinical Electroencephalography 17: 176–180. 3791644

[hbm70385-bib-0012] Beste, C. , M. Humphries , and C. Saft . 2014. “Striatal Disorders Dissociate Mechanisms of Enhanced and Impaired Response Selection ‐ Evidence From Cognitive Neurophysiology and Computational Modelling.” Neuroimage Clinical 4: 623–634. 10.1016/j.nicl.2014.04.003. 24936413 PMC4053645

[hbm70385-bib-0013] Beste, C. , C. K. E. Moll , M. Pötter‐Nerger , and A. Münchau . 2018. “Striatal Microstructure and Its Relevance for Cognitive Control.” Trends in Cognitive Sciences 22: 747–751. 10.1016/j.tics.2018.06.007. 30017252

[hbm70385-bib-0014] Beste, C. , A. Münchau , and C. Frings . 2023. “Towards a Systematization of Brain Oscillatory Activity in Actions.” Communications Biology 6: 137. 10.1038/s42003-023-04531-9. 36732548 PMC9894929

[hbm70385-bib-0015] Beste, C. , and C. Saft . 2015. “Action Selection in a Possible Model of Striatal Medium Spiny Neuron Dysfunction: Behavioral and EEG Data in a Patient With Benign Hereditary Chorea.” Brain Structure & Function 220: 221–228. 10.1007/s00429-013-0649-9. 24135770

[hbm70385-bib-0016] Beste, C. , C. Saft , O. Güntürkün , and M. Falkenstein . 2008. “Increased Cognitive Functioning in Symptomatic Huntington's Disease as Revealed by Behavioral and Event‐Related Potential Indices of Auditory Sensory Memory and Attention.” Journal of Neuroscience 28: 11695–11702. 10.1523/JNEUROSCI.2659-08.2008. 18987205 PMC6671311

[hbm70385-bib-0017] Bigdely‐Shamlo, N. , T. Mullen , C. Kothe , K.‐M. Su , and K. A. Robbins . 2015. “The PREP Pipeline: Standardized Preprocessing for Large‐Scale EEG Analysis.” Frontiers in Neuroinformatics 9: 16. 10.3389/fninf.2015.00016. 26150785 PMC4471356

[hbm70385-bib-0018] Botvinick, M. M. 2007. “Conflict Monitoring and Decision Making: Reconciling Two Perspectives on Anterior Cingulate Function.” Cognitive, Affective, & Behavioral Neuroscience 7: 356–366. 10.3758/CABN.7.4.356. 18189009

[hbm70385-bib-0019] Botvinick, M. M. , J. D. Cohen , and C. S. Carter . 2004. “Conflict Monitoring and Anterior Cingulate Cortex: an Update.” Trends in Cognitive Sciences 8: 539–546. 10.1016/j.tics.2004.10.003. 15556023

[hbm70385-bib-0020] Boy, F. , C. J. Evans , R. A. E. Edden , K. D. Singh , M. Husain , and P. Sumner . 2010. “Individual Differences in Subconscious Motor Control Predicted by GABA Concentration in SMA.” Current Biology 20: 1779–1785. 10.1016/j.cub.2010.09.003. 20888227 PMC3128986

[hbm70385-bib-0021] Boy, F. , M. Husain , K. D. Singh , and P. Sumner . 2010. “Supplementary Motor Area Activations in Unconscious Inhibition of Voluntary Action.” Experimental Brain Research 206: 441–448. 10.1007/s00221-010-2417-x. 20871983

[hbm70385-bib-0022] Calderon, C. B. , F. van Opstal , P. Peigneux , T. Verguts , and W. Gevers . 2018. “Task‐Relevant Information Modulates Primary Motor Cortex Activity Before Movement Onset.” Frontiers in Human Neuroscience 12: 93. 10.3389/fnhum.2018.00093. 29593518 PMC5861186

[hbm70385-bib-0023] Carrey, N. , F. P. MacMaster , S. J. Sparkes , S. C. Khan , and V. Kusumakar . 2002. “Glutamatergic Changes With Treatment in Attention Deficit Hyperactivity Disorder: A Preliminary Case Series.” Journal of Child and Adolescent Psychopharmacology 12: 331–336. 10.1089/104454602762599871. 12625993

[hbm70385-bib-0024] Cavanagh, J. F. , and M. J. Frank . 2014. “Frontal Theta as a Mechanism for Cognitive Control.” Trends in Cognitive Sciences 18: 414–421. 10.1016/j.tics.2014.04.012. 24835663 PMC4112145

[hbm70385-bib-0025] Challman, T. D. , and J. J. Lipsky . 2000. “Methylphenidate: Its Pharmacology and Uses.” Mayo Clinic Proceedings 75: 711–721. 10.4065/75.7.711. 10907387

[hbm70385-bib-0026] Cheng, J. , Z. Xiong , L. J. Duffney , et al. 2014. “Methylphenidate Exerts Dose‐Dependent Effects on Glutamate Receptors and Behaviors. Biological Psychiatry, N‐Methyl‐D‐Aspartate Receptors, Synaptic Plasticity, Psychopathology, and Treatment.” Biological Psychiatry 76: 953–962. 10.1016/j.biopsych.2014.04.003. 24832867 PMC4194277

[hbm70385-bib-0027] Coull, J. T. , F. Vidal , and B. Burle . 2016. “When to Act, or Not to Act: That's the SMA'S Question.” Current Opinion in Behavioral Sciences, Time in Perception and Action 8: 14–21. 10.1016/j.cobeha.2016.01.003.

[hbm70385-bib-0028] Deiber, M.‐P. , E. Sallard , C. Ludwig , C. Ghezzi , J. Barral , and V. Ibanez . 2012. “EEG Alpha Activity Reflects Motor Preparation Rather Than the Mode of Action Selection.” Frontiers in Integrative Neuroscience 6: 59. 10.3389/fnint.2012.00059. 22912607 PMC3418545

[hbm70385-bib-0029] Dipasquale, O. , D. Martins , A. Sethi , et al. 2020. “Unravelling the Effects of Methylphenidate on the Dopaminergic and Noradrenergic Functional Circuits.” Neuropsychopharmacology 45: 1482–1489. 10.1038/s41386-020-0724-x. 32473593 PMC7360745

[hbm70385-bib-0030] Diveica, V. , M. C. Riedel , T. Salo , A. R. Laird , R. L. Jackson , and R. J. Binney . 2023. “Graded Functional Organization in the Left Inferior Frontal Gyrus: Evidence From Task‐Free and Task‐Based Functional Connectivity.” Cerebral Cortex 33: 11384–11399. 10.1093/cercor/bhad373. 37833772 PMC10690868

[hbm70385-bib-0031] Dockree, P. M. , J. J. Barnes , N. Matthews , et al. 2017. “The Effects of Methylphenidate on the Neural Signatures of Sustained Attention.” Biological Psychiatry 82: 687–694. 10.1016/j.biopsych.2017.04.016. 28599833

[hbm70385-bib-0032] Duda, J. M. , A. D. Moser , C. S. Zuo , et al. 2021. “Repeatability and Reliability of GABA Measurements With Magnetic Resonance Spectroscopy in Healthy Young Adults.” Magnetic Resonance in Medicine 85: 2359–2369. 10.1002/mrm.28587. 33216412 PMC7902337

[hbm70385-bib-0033] Dydak, U. , Y.‐M. Jiang , L.‐L. Long , et al. 2011. “In Vivo Measurement of Brain GABA Concentrations by Magnetic Resonance Spectroscopy in Smelters Occupationally Exposed to Manganese.” Environmental Health Perspectives 119: 219–224. 10.1289/ehp.1002192. 20876035 PMC3040609

[hbm70385-bib-0034] Erlij, D. , J. Acosta‐García , M. Rojas‐Márquez , et al. 2012. “Dopamine D4 Receptor Stimulation in GABAergic Projections of the Globus Pallidus to the Reticular Thalamic Nucleus and the Substantia Nigra Reticulata of the Rat Decreases Locomotor Activity.” Neuropharmacology 62: 1111–1118. 10.1016/j.neuropharm.2011.11.001. 22108379

[hbm70385-bib-0035] Ester, M. , H.‐P. Kriegel , J. Sander , and X. Xu . 1996. “A Density‐Based Algorithm for Discovering Clusters in Large Spatial Databases With Noise.” In Proceedings of the Second International Conference on Knowledge Discovery and Data Mining, vol. 96, 226–231. ACM.

[hbm70385-bib-0036] Farias, T. L. , V. Marinho , V. Carvalho , et al. 2019. “Methylphenidate Modifies Activity in the Prefrontal and Parietal Cortex Accelerating the Time Judgment.” Neurological Sciences 40: 829–837. 10.1007/s10072-018-3699-1. 30693423

[hbm70385-bib-0037] Faul, F. , E. Erdfelder , A.‐G. Lang , and A. Buchner . 2007. “G*Power 3: A Flexible Statistical Power Analysis Program for the Social, Behavioral, and Biomedical Sciences.” Behavior Research Methods 39: 175–191. 10.3758/BF03193146. 17695343

[hbm70385-bib-0038] Fisher, R. A. 1921. “014: ‘On the Probable Error’ of a Coefficient of Correlation Deduced from a Small Sample.”

[hbm70385-bib-0039] Foxe, J. J. , and A. C. Snyder . 2011. “The Role of Alpha‐Band Brain Oscillations as a Sensory Suppression Mechanism During Selective Attention.” Frontiers in Psychology 2: 154. 10.3389/fpsyg.2011.00154. 21779269 PMC3132683

[hbm70385-bib-0040] Freese, L. , E. J. Muller , M. F. Souza , et al. 2012. “GABA System Changes in Methylphenidate Sensitized Female Rats.” Behavioural Brain Research 231: 181–186. 10.1016/j.bbr.2012.03.017. 22460063

[hbm70385-bib-0041] Fujihara, K. , K. Narita , Y. Suzuki , et al. 2015. “Relationship of γ‐Aminobutyric Acid and Glutamate + Glutamine Concentrations in the Perigenual Anterior Cingulate Cortex With Performance of Cambridge Gambling Task.” NeuroImage 109: 102–108. 10.1016/j.neuroimage.2015.01.014. 25583607

[hbm70385-bib-0042] García‐Pérez, M. A. 2023. “Use and Misuse of Corrections for Multiple Testing.” Methods in Psychology 8: 100120. 10.1016/j.metip.2023.100120.

[hbm70385-bib-0043] Govindaraju, V. , K. Young , and A. A. Maudsley . 2000. “Proton NMR Chemical Shifts and Coupling Constants for Brain Metabolites.” NMR in Biomedicine 13: 129–153. 10.1002/1099-1492(200005)13:3<129::AID-NBM619>3.0.CO;2-V. 10861994

[hbm70385-bib-0044] Gross, J. , J. Kujala , M. Hämäläinen , L. Timmermann , A. Schnitzler , and R. Salmelin . 2001. “Dynamic Imaging of Coherent Sources: Studying Neural Interactions in the Human Brain.” Proceedings of the National Academy of Sciences 98: 694–699. 10.1073/pnas.98.2.694. PMC14650 11209067

[hbm70385-bib-0045] Haag, L. , C. Quetscher , S. Dharmadhikari , U. Dydak , T. Schmidt‐Wilcke , and C. Beste . 2015. “Interrelation of Resting State Functional Connectivity, Striatal GABA Levels, and Cognitive Control Processes.” Human Brain Mapping 36: 4383–4393. 10.1002/hbm.22920. 26354091 PMC6868959

[hbm70385-bib-0046] Hodzhev, Y. , J. Yordanova , M. Diruf , et al. 2012. “Methylphenidate (MPH) Promotes Visual Cortical Activation in Healthy Adults in a Cued Visuomotor Task.” Journal of Neural Transmission 119: 1455–1464. 10.1007/s00702-012-0799-6. 22460297

[hbm70385-bib-0047] Humphries, M. D. , R. D. Stewart , and K. N. Gurney . 2006. “A Physiologically Plausible Model of Action Selection and Oscillatory Activity in the Basal Ganglia.” Journal of Neuroscience 26: 12921–12942. 10.1523/JNEUROSCI.3486-06.2006. 17167083 PMC6674973

[hbm70385-bib-0048] Ivanov, I. , X. Liu , S. Clerkin , et al. 2014. “Methylphenidate and Brain Activity in a Reward/Conflict Paradigm: Role of the Insula in Task Performance.” European Neuropsychopharmacology 24: 897–906. 10.1016/j.euroneuro.2014.01.017. 24491951

[hbm70385-bib-0049] Jensen, O. , and A. Mazaheri . 2010. “Shaping Functional Architecture by Oscillatory Alpha Activity: Gating by Inhibition.” Frontiers in Human Neuroscience 4: 186. 10.3389/fnhum.2010.00186. 21119777 PMC2990626

[hbm70385-bib-0050] Kaiser, L. G. , K. Young , and G. B. Matson . 2008. “Numerical Simulations of Localized High Field 1H MR Spectroscopy.” Journal of Magnetic Resonance 195: 67–75. 10.1016/j.jmr.2008.08.010. 18789736 PMC2585774

[hbm70385-bib-0051] Klimesch, W. 2012. “Alpha‐Band Oscillations, Attention, and Controlled Access to Stored Information.” Trends in Cognitive Sciences 16: 606–617. 10.1016/j.tics.2012.10.007. 23141428 PMC3507158

[hbm70385-bib-0052] Klimesch, W. , P. Sauseng , and S. Hanslmayr . 2007. “EEG Alpha Oscillations: The Inhibition–Timing Hypothesis.” Brain Research Reviews 53: 63–88. 10.1016/j.brainresrev.2006.06.003. 16887192

[hbm70385-bib-0053] Koyun, A. H. , N. Talebi , A. Werner , et al. 2024. “Interactions of Catecholamines and GABA+ in Cognitive Control: Insights From EEG and 1H‐MRS.” NeuroImage 293: 120619. 10.1016/j.neuroimage.2024.120619. 38679186

[hbm70385-bib-0054] Koyun, A. H. , P. Wendiggensen , V. Roessner , C. Beste , and A.‐K. Stock . 2024. “Effects of Catecholaminergic and Transcranial Direct Current Stimulation on Response Inhibition.” International Journal of Neuropsychopharmacology 27: pyae023. 10.1093/ijnp/pyae023. 38742426 PMC11184454

[hbm70385-bib-0055] Koyun, A. H. , A. Werner , P. Kuntke , V. Roessner , C. Beste , and A.‐K. Stock . 2025. “GABA and Glx Levels in Cortico‐Subcortical Networks Predict Catecholaminergic Effects on Response Inhibition.” Journal of Psychopharmacology 39, no. 8: 769–781. 10.1177/02698811251340893. 40539252 PMC12287562

[hbm70385-bib-0056] Kreis, R. , and C. S. Bolliger . 2012. “The Need for Updates of Spin System Parameters, Illustrated for the Case of γ‐Aminobutyric Acid.” NMR in Biomedicine 25: 1401–1403. 10.1002/nbm.2810. 22585581

[hbm70385-bib-0057] Linssen, A. M. W. , A. Sambeth , E. F. P. M. Vuurman , and W. J. Riedel . 2014. “Cognitive Effects of Methylphenidate in Healthy Volunteers: a Review of Single Dose Studies.” International Journal of Neuropsychopharmacology 17: 961–977. 10.1017/S1461145713001594. 24423151

[hbm70385-bib-0058] Manz, K. M. , B. C. Coleman , C. A. Grueter , B. C. Shields , M. R. Tadross , and B. A. Grueter . 2021. “Noradrenergic Signaling Disengages Feedforward Transmission in the Nucleus Accumbens Shell.” Journal of Neuroscience 41: 3752–3763. 10.1523/JNEUROSCI.2420-20.2021. 33737458 PMC8084318

[hbm70385-bib-0059] Marjańska, M. , S. Lehéricy , R. Valabrègue , et al. 2013. “Brain Dynamic Neurochemical Changes in Dystonic Patients: a Magnetic Resonance Spectroscopy Study.” Movement Disorders 28: 201–209. 10.1002/mds.25279. 23239076 PMC4410978

[hbm70385-bib-0060] Mikkelsen, M. , P. B. Barker , P. K. Bhattacharyya , et al. 2017. “Big GABA: Edited MR Spectroscopy at 24 Research Sites.” NeuroImage 159: 32–45. 10.1016/j.neuroimage.2017.07.021. 28716717 PMC5700835

[hbm70385-bib-0061] Mikkelsen, M. , D. L. Rimbault , P. B. Barker , et al. 2019. “Big GABA II: Water‐Referenced Edited MR Spectroscopy at 25 Research Sites.” NeuroImage 191: 537–548. 10.1016/j.neuroimage.2019.02.059. 30840905 PMC6818968

[hbm70385-bib-0062] Moss, H. E. , S. Abdallah , P. Fletcher , et al. 2005. “Selecting Among Competing Alternatives: Selection and Retrieval in the Left Inferior Frontal Gyrus.” Cerebral Cortex 15: 1723–1735. 10.1093/cercor/bhi049. 15728742 PMC3838943

[hbm70385-bib-0063] Mückschel, M. , G. Dippel , and C. Beste . 2017. “Distinguishing Stimulus and Response Codes in Theta Oscillations in Prefrontal Areas During Inhibitory Control of Automated Responses.” Human Brain Mapping 38: 5681–5690. 10.1002/hbm.23757. 28782869 PMC6867003

[hbm70385-bib-0064] Mückschel, M. , K. Gohil , T. Ziemssen , and C. Beste . 2017. “The Norepinephrine System and Its Relevance for Multi‐Component Behavior.” NeuroImage 146: 1062–1070. 10.1016/j.neuroimage.2016.10.007. 27720820

[hbm70385-bib-0065] Mückschel, M. , A.‐K. Stock , G. Dippel , W. Chmielewski , and C. Beste . 2016. “Interacting Sources of Interference During Sensorimotor Integration Processes.” NeuroImage 125: 342–349. 10.1016/j.neuroimage.2015.09.075. 26596550

[hbm70385-bib-0066] Nachev, P. , G. Rees , A. Parton , C. Kennard , and M. Husain . 2005. “Volition and Conflict in Human Medial Frontal Cortex.” Current Biology 15: 122–128. 10.1016/j.cub.2005.01.006. 15668167 PMC2648721

[hbm70385-bib-0067] Near, J. , C. J. Evans , N. A. J. Puts , P. B. Barker , and R. A. E. Edden . 2013. “J‐Difference Editing of Gamma‐Aminobutyric Acid (GABA): Simulated and Experimental Multiplet Patterns.” Magnetic Resonance in Medicine 70: 1183–1191. 10.1002/mrm.24572. 23213033 PMC3601567

[hbm70385-bib-0068] Pape, A.‐A. , and M. Siegel . 2016. “Motor Cortex Activity Predicts Response Alternation During Sensorimotor Decisions.” Nature Communications 7: 13098. 10.1038/ncomms13098. PMC5059771 27713396

[hbm70385-bib-0069] Parra, L. C. , C. D. Spence , A. D. Gerson , and P. Sajda . 2005. “Recipes for the Linear Analysis of EEG.” NeuroImage 28: 326–341. 10.1016/j.neuroimage.2005.05.032. 16084117

[hbm70385-bib-0070] Pauls, A. M. , O. G. O'Daly , K. Rubia , W. J. Riedel , S. C. R. Williams , and M. A. Mehta . 2012. “Methylphenidate Effects on Prefrontal Functioning During Attentional‐Capture and Response Inhibition.” Biological Psychiatry 72: 142–149. 10.1016/j.biopsych.2012.03.028. 22552046

[hbm70385-bib-0071] Peek, A. L. , T. J. Rebbeck , A. M. Leaver , et al. 2023. “A Comprehensive Guide to MEGA‐PRESS for GABA Measurement.” Analytical Biochemistry 669: 115113. 10.1016/j.ab.2023.115113. 36958511 PMC10805000

[hbm70385-bib-0072] Petzold, J. , A. Kienast , Y. Lee , et al. 2019. “Baseline Impulsivity May Moderate L‐DOPA Effects on Value‐Based Decision‐Making.” Scientific Reports 9: 5652. 10.1038/s41598-019-42124-x. 30948756 PMC6449394

[hbm70385-bib-0073] Plenz, D. 2003. “When Inhibition Goes Incognito: Feedback Interaction Between Spiny Projection Neurons in Striatal Function.” Trends in Neurosciences 26: 436–443. 10.1016/S0166-2236(03)00196-6. 12900175

[hbm70385-bib-0074] Prochnow, A. , M. Mückschel , E. Eggert , et al. 2024. “The Ability to Voluntarily Regulate Theta Band Activity Affects How Pharmacological Manipulation of the Catecholaminergic System Impacts Cognitive Control.” International Journal of Neuropsychopharmacology 27: pyae003. 10.1093/ijnp/pyae003. 38181228 PMC10810285

[hbm70385-bib-0075] Quetscher, C. , A. Yildiz , S. Dharmadhikari , et al. 2015. “Striatal GABA‐MRS Predicts Response Inhibition Performance and Its Cortical Electrophysiological Correlates.” Brain Structure & Function 220: 3555–3564. 10.1007/s00429-014-0873-y. 25156575 PMC4447607

[hbm70385-bib-0076] Redgrave, P. , N. Vautrelle , and J. N. J. Reynolds . 2011. “Functional Properties of the Basal Ganglia's Re‐Entrant Loop Architecture: Selection and Reinforcement.” Neuroscience 198: 138–151. 10.1016/j.neuroscience.2011.07.060. 21821101

[hbm70385-bib-0077] Rhodes, E. , W. C. Gaetz , J. Marsden , and S. D. Hall . 2018. “Transient Alpha and Beta Synchrony Underlies Preparatory Recruitment of Directional Motor Networks.” Journal of Cognitive Neuroscience 30: 867–875. 10.1162/jocn_a_01250. 29488848

[hbm70385-bib-0078] Rickham, P. P. 1964. “Human Experimentation. Code of Ethics of the World Medical Association. Declaration of Helsinki.” British Medical Journal 2: 177. 10.1136/bmj.2.5402.177. 14150898 PMC1816102

[hbm70385-bib-0079] Robbins, T. W. , and A. F. T. Arnsten . 2009. “The Neuropsychopharmacology of Fronto‐Executive Function: Monoaminergic Modulation.” Annual Review of Neuroscience 32: 267–287. 10.1146/annurev.neuro.051508.135535. PMC2863127 19555290

[hbm70385-bib-0080] Rostron, C. L. , E. Kaplan , V. Gaeta , R. Nigriello , and E. J. Dommett . 2013. “The Effects of Methylphenidate on Cognitive Performance of Healthy Male Rats.” Frontiers in Neuroscience 7: 97. 10.3389/fnins.2013.00097. 23781167 PMC3680706

[hbm70385-bib-0081] Rubia, K. , R. Halari , A.‐M. Mohammad , E. Taylor , and M. Brammer . 2011. “Methylphenidate Normalizes Frontocingulate Underactivation During Error Processing in Attention‐Deficit/Hyperactivity Disorder.” Biological Psychiatry 70: 255–262. 10.1016/j.biopsych.2011.04.018. 21664605 PMC3139835

[hbm70385-bib-0082] Sadaghiani, S. , and A. Kleinschmidt . 2016. “Brain Networks and α‐Oscillations: Structural and Functional Foundations of Cognitive Control.” Trends in Cognitive Sciences 20: 805–817. 10.1016/j.tics.2016.09.004. 27707588

[hbm70385-bib-0083] Shungu, D. C. , X. Mao , R. Gonzales , et al. 2016. “Brain γ‐Aminobutyric Acid (GABA) Detection in Vivo With the J‐Editing (1) H MRS Technique: a Comprehensive Methodological Evaluation of Sensitivity Enhancement, Macromolecule Contamination and Test‐Retest Reliability.” NMR in Biomedicine 29: 932–942. 10.1002/nbm.3539. 27173449 PMC4909570

[hbm70385-bib-0084] Silveri, M. M. , J. T. Sneider , D. J. Crowley , et al. 2013. “Frontal Lobe γ‐Aminobutyric Acid Levels During Adolescence: Associations With Impulsivity and Response Inhibition.” Biological Psychiatry 74: 296–304. 10.1016/j.biopsych.2013.01.033. 23498139 PMC3695052

[hbm70385-bib-0085] Spencer, R. C. , R. M. Klein , and C. W. Berridge . 2012. “Psychostimulants Act Within the Prefrontal Cortex to Improve Cognitive Function.” Biological Psychiatry 72: 221–227. 10.1016/j.biopsych.2011.12.002. 22209638 PMC3319517

[hbm70385-bib-0086] Stock, A.‐K. , J. Friedrich , and C. Beste . 2016. “Subliminally and Consciously Induced Cognitive Conflicts Interact at Several Processing Levels.” Cortex 85: 75–89. 10.1016/j.cortex.2016.09.027. 27838544

[hbm70385-bib-0087] Stock, A.‐K. , A. Werner , P. Kuntke , et al. 2023. “GABA and Glutamate Concentrations in the Striatum and Anterior Cingulate Cortex Not Found to Be Associated With Cognitive Flexibility.” Brain Sciences 13: 1192. 10.3390/brainsci13081192. 37626548 PMC10452168

[hbm70385-bib-0088] Swick, D. , V. Ashley , and A. U. Turken . 2008. “Left Inferior Frontal Gyrus Is Critical for Response Inhibition.” BMC Neuroscience 9: 102. 10.1186/1471-2202-9-102. 18939997 PMC2588614

[hbm70385-bib-0089] Takacs, A. , A.‐K. Stock , P. Kuntke , A. Werner , and C. Beste . 2021. “On the Functional Role of Striatal and Anterior Cingulate GABA+ in Stimulus‐Response Binding.” Human Brain Mapping 42: 1863–1878. 10.1002/hbm.25335. 33421290 PMC7978129

[hbm70385-bib-0090] Takei, Y. , K. Fujihara , M. Tagawa , et al. 2016. “The Inhibition/Excitation Ratio Related to Task‐Induced Oscillatory Modulations During a Working Memory Task: A Multtimodal‐Imaging Study Using MEG and MRS.” NeuroImage 128: 302–315. 10.1016/j.neuroimage.2015.12.057. 26780573

[hbm70385-bib-0091] Tomkins, A. , E. Vasilaki , C. Beste , K. Gurney , and M. D. Humphries . 2013. “Transient and Steady‐State Selection in the Striatal Microcircuit.” Frontiers in Computational Neuroscience 7: 192. 10.3389/fncom.2013.00192. 24478684 PMC3895806

[hbm70385-bib-0092] Tremblay, S. , V. Beaulé , S. Proulx , et al. 2014. “The Use of Magnetic Resonance Spectroscopy as a Tool for the Measurement of Bi‐Hemispheric Transcranial Electric Stimulation Effects on Primary Motor Cortex Metabolism.” Journal of Visualized Experiments: e51631. 10.3791/51631. 25490453 PMC4354197

[hbm70385-bib-0093] Tritsch, N. X. , and B. L. Sabatini . 2012. “Dopaminergic Modulation of Synaptic Transmission in Cortex and Striatum.” Neuron 76: 33–50. 10.1016/j.neuron.2012.09.023. 23040805 PMC4386589

[hbm70385-bib-0094] Tzourio‐Mazoyer, N. , B. Landeau , D. Papathanassiou , et al. 2002. “Automated Anatomical Labeling of Activations in SPM Using a Macroscopic Anatomical Parcellation of the MNI MRI Single‐Subject Brain.” NeuroImage 15: 273–289. 10.1006/nimg.2001.0978. 11771995

[hbm70385-bib-0095] Urban, K. R. , Y.‐C. Li , and W.‐J. Gao . 2013. “Treatment With a Clinically‐Relevant Dose of Methylphenidate Alters NMDA Receptor Composition and Synaptic Plasticity in the Juvenile Rat Prefrontal Cortex.” Neurobiology of Learning and Memory 101: 65–74. 10.1016/j.nlm.2013.01.004. 23333502 PMC3602399

[hbm70385-bib-0096] Weaver, B. , and K. L. Wuensch . 2013. “SPSS and SAS Programs for Comparing Pearson Correlations and OLS Regression Coefficients.” Behavior Research 45: 880–895. 10.3758/s13428-012-0289-7. 23344734

[hbm70385-bib-0097] Wendiggensen, P. , F. Ghin , A. H. Koyun , A.‐K. Stock , and C. Beste . 2022. “Pretrial Theta Band Activity Affects Context‐Dependent Modulation of Response Inhibition.” Journal of Cognitive Neuroscience 34: 605–617. 10.1162/jocn_a_01816. 35061021

[hbm70385-bib-0098] Whissell, P. D. , J. D. Cajanding , N. Fogel , and J. C. Kim . 2015. “Comparative Density of CCK‐ and PV‐GABA Cells Within the Cortex and Hippocampus.” Frontiers in Neuroanatomy 9: 124. 10.3389/fnana.2015.00124. 26441554 PMC4585045

[hbm70385-bib-0099] Willemssen, R. , M. Falkenstein , M. Schwarz , T. Müller , and C. Beste . 2011. “Effects of Aging, Parkinson's Disease, and Dopaminergic Medication on Response Selection and Control.” Neurobiology of Aging 32: 327–335. 10.1016/j.neurobiolaging.2009.02.002. 19269061

[hbm70385-bib-0100] Winkler, I. , S. Haufe , and M. Tangermann . 2011. “Automatic Classification of Artifactual ICA‐Components for Artifact Removal in EEG Signals.” Behavioral and Brain Functions 7: 30. 10.1186/1744-9081-7-30. 21810266 PMC3175453

[hbm70385-bib-0101] Yildiz, A. , C. Quetscher , S. Dharmadhikari , et al. 2014. “Feeling Safe in the Plane: Neural Mechanisms Underlying Superior Action Control in Airplane Pilot Trainees—A Combined EEG/MRS Study.” Human Brain Mapping 35: 5040–5051. 10.1002/hbm.22530. 24753040 PMC4452896

[hbm70385-bib-0102] Zhang, J. X. , C.‐M. Feng , P. T. Fox , J.‐H. Gao , and L. H. Tan . 2004. “Is Left Inferior Frontal Gyrus a General Mechanism for Selection?” NeuroImage 23: 596–603. 10.1016/j.neuroimage.2004.06.006. 15488409

