# Supplementary material for: Dose‐Dependent Effects of Catecholaminergic Modulation on Interference Control: Role of Baseline GABA and Glx in Cortico‐Subcortical Networks
Source: Hum Brain Mapp. 2025 Oct 23;46(15):e70385. doi: 10.1002/hbm.70385 (PMC12547842; doi:10.1002/hbm.70385)
Supplement: Supplementary file 1 — Data S1: Supporting Information. [file HBM-46-e70385-s001.pdf]

Supplementary materials

Publication title:	***Dose-Dependent Effects of Catecholaminergic Modulation on Interference Control: Role of Baseline GABA and Glx in Cortico-Subcortical Networks***
Authors:	*Anna Helin Koyun, Annett Werner, Paul Kuntke, Veit Rößner, Christian Beste, Ann-Kathrin Stock*

ANOVA outcomes and descriptive data for each experimental condition and (sub)group

Please refer for the following pages for the full outcomes of each ANOVA (*Tables S-1 and S-2* for the accuracy ANOVA and *Tables S-4 and S-5* for the hit response times ANOVA) and tables providing descriptive data (sample size n, mean and standard error of the mean/SEM) for accuracy (*Table S-3*) and for hit response times (*Table S-6*).

Table S-1: Within subject effects of the ANOVA for accuracy.

Cases	Sum of Squares	df	Mean Square	F	p	η^2_p
MPH/Placebo	93.263	1	93.263	2.115	0.150	0.029
MPH/Placebo ✻ MPH dosage group	74.859	1	74.859	1.698	0.197	0.023
MPH/Placebo ✻ Order of drug administration	762.264	1	762.264	17.287	< .001	0.194
MPH/Placebo ✻ MPH dosage group ✻ Order of drug administration	86.690	1	86.690	1.966	0.165	0.027
Residuals	3174.732	72	44.094			
Prime	9411.180	1	9411.180	69.101	< .001	0.490
Prime ✻ MPH dosage group	160.178	1	160.178	1.176	0.282	0.016
Prime ✻ Order of drug administration	1.523	1	1.523	0.011	0.916	1.553×10^{-4}
Prime ✻ MPH dosage group ✻ Order of drug administration	57.221	1	57.221	0.420	0.519	0.006
Residuals	9806.020	72	136.195			
Flanker	3918.783	1	3918.783	84.781	< .001	0.541
Flanker ✻ MPH dosage group	13.922	1	13.922	0.301	0.585	0.004
Flanker ✻ Order of drug administration	45.414	1	45.414	0.983	0.325	0.013
Flanker ✻ MPH dosage group ✻ Order of drug administration	18.808	1	18.808	0.407	0.526	0.006
Residuals	3328.024	72	46.223			
MPH/Placebo ✻ Prime	7.085	1	7.085	0.265	0.608	0.004
MPH/Placebo ✻ Prime ✻ MPH dosage group	108.863	1	108.863	4.077	0.047	0.054
MPH/Placebo ✻ Prime ✻ Order of drug administration	159.032	1	159.032	5.956	0.017	0.076
MPH/Placebo ✻ Prime ✻ MPH dosage group ✻ Order of drug administration	63.735	1	63.735	2.387	0.127	0.032
Residuals	1922.404	72	26.700			
MPH/Placebo ✻ Flanker	26.480	1	26.480	3.324	0.072	0.044

Cases	Sum of Squares	df	Mean Square	F	p	η^2_p
MPH/Placebo ✻ Flanker ✻ MPH dosage group	1.638	1	1.638	0.206	0.652	0.003
MPH/Placebo ✻ Flanker ✻ Order of drug administration	48.798	1	48.798	6.126	0.016	0.078
MPH/Placebo ✻ Flanker ✻ MPH dosage group ✻ Order of drug administration	27.335	1	27.335	3.432	0.068	0.045
Residuals	573.506	72	7.965			
Prime ✻ Flanker	805.848	1	805.848	55.042	< .001	0.433
Prime ✻ Flanker ✻ MPH dosage group	58.912	1	58.912	4.024	0.049	0.053
Prime ✻ Flanker ✻ Order of drug administration	5.653	1	5.653	0.386	0.536	0.005
Prime ✻ Flanker ✻ MPH dosage group ✻ Order of drug administration	2.496	1	2.496	0.170	0.681	0.002
Residuals	1054.121	72	14.641			
MPH/Placebo ✻ Prime ✻ Flanker	2.665	1	2.665	0.233	0.631	0.003
MPH/Placebo ✻ Prime ✻ Flanker ✻ MPH dosage group	6.198	1	6.198	0.542	0.464	0.007
MPH/Placebo ✻ Prime ✻ Flanker ✻ Order of drug administration	0.099	1	0.099	0.009	0.926	1.199×10^{-4}
MPH/Placebo ✻ Prime ✻ Flanker ✻ MPH dosage group ✻ Order of drug administration	2.867	1	2.867	0.251	0.618	0.003
Residuals	823.737	72	11.441			

Note. Type III Sum of Squares. Significant effects are highlighted in blue font. The significant effects that are underscored were reported in the main manuscript and further analyzed with post hoc tests. Please note that the decision to not report some of the effects (those are not underscored) was based on either the fact that there were higher-level interactions that comprised additional factors, rendering the insights gained from those lower-level significances irrelevant, or on the fact that those interactions did not comprise the relevant task effect (i.e., the interaction of prime * flanker) or the factor of MPH dosage group, which were central to our research question.
.

Table S-2: Between subject effects of the ANOVA for accuracy.

Cases	Sum of Squares	df	Mean Square	F	p	η^2_p
MPH dosage group	31.711	1	31.711	0.127	0.722	0.002
Order of drug administration	9.167	1	9.167	0.037	0.848	5.112×10^{-4}
MPH dosage group ∗ Order of drug administration	191.898	1	191.898	0.771	0.383	0.011
Residuals	17922.128	72	248.918			

Note. Type III Sum of Squares. Significant effects are highlighted in blue font.

Table S-3: Descriptive data for accuracy in percent (%).

MPH/Placebo	Prime Compatibility	Flanker Congruency	MPH Dosage Group	Order of Drug Administration	N	Mean (%)	SEM
Placebo	Incompatible	Incongruent	0.25 mg/kg	MPH first	17	86.713	3.091
				MPH second	21	82.986	2.617
			0.50 mg/kg	MPH first	20	87.015	2.985
				MPH second	18	83.797	3.733
		Congruent	0.25 mg/kg	MPH first	17	95.521	1.282
				MPH second	21	91.336	2.149
			0.50 mg/kg	MPH first	20	94.417	1.471
				MPH second	18	91.066	2.142
	Compatible	Incongruent	0.25 mg/kg	MPH first	17	96.956	0.700
				MPH second	21	95.061	1.206
			0.50 mg/kg	MPH first	20	95.966	1.229
				MPH second	18	94.752	1.186
		Congruent	0.25 mg/kg	MPH first	17	99.680	0.124
				MPH second	21	98.034	0.550
			0.50 mg/kg	MPH first	20	99.008	0.279
				MPH second	18	98.297	0.453
MPH	Incompatible	Incongruent	0.25 mg/kg	MPH first	17	84.510	3.535
				MPH second	21	84.577	2.973
			0.50 mg/kg	MPH first	20	84.591	3.088
				MPH second	18	93.050	1.914
		Congruent	0.25 mg/kg	MPH first	17	92.890	2.134
				MPH second	21	92.375	2.370
			0.50 mg/kg	MPH first	20	92.591	1.701
				MPH second	18	96.288	1.011
	Compatible	Incongruent	0.25 mg/kg	MPH first	17	96.687	1.101
				MPH second	21	97.528	0.787
			0.50 mg/kg	MPH first	20	94.419	1.678
				MPH second	18	97.523	0.742
		Congruent	0.25 mg/kg	MPH first	17	99.152	0.391
				MPH second	21	99.221	0.283
			0.50 mg/kg	MPH first	20	98.747	0.290
				MPH second	18	99.032	0.554

Table S-4: Within subject effects of the ANOVA for hit response times.

Cases	Sum of Squares	df	Mean Square	F	p	η^2_p
MPH/Placebo	6338.465	1	6338.465	7.067	0.010	0.089
MPH/Placebo ✻ MPH dosage group	81.633	1	81.633	0.091	0.764	0.001
MPH/Placebo ✻ Order of drug administration	129.993	1	129.993	0.145	0.705	0.002
MPH/Placebo ✻ MPH dosage group ✻ Order of drug administration	2203.809	1	2203.809	2.457	0.121	0.033
Residuals	64574.304	72	896.865			
Prime	276886.248	1	276886.248	238.982	< .001	0.768
Prime ✻ MPH dosage group	6543.304	1	6543.304	5.648	0.020	0.073
Prime ✻ Order of drug administration	0.021	1	0.021	1.845×10^{-5}	0.997	2.562×10^{-7}
Prime ✻ MPH dosage group ✻ Order of drug administration	5.789	1	5.789	0.005	0.944	6.939×10^{-5}
Residuals	83419.641	72	1158.606			
Flanker	65251.060	1	65251.060	302.956	< .001	0.808
Flanker ✻ MPH dosage group	168.641	1	168.641	0.783	0.379	0.011
Flanker ✻ Order of drug administration	500.595	1	500.595	2.324	0.132	0.031
Flanker ✻ MPH dosage group ✻ Order of drug administration	59.388	1	59.388	0.276	0.601	0.004
Residuals	15507.466	72	215.381			
MPH/Placebo ✻ Prime	114.654	1	114.654	0.470	0.495	0.006
MPH/Placebo ✻ Prime ✻ MPH dosage group	1862.024	1	1862.024	7.630	0.007	0.096
MPH/Placebo ✻ Prime ✻ Order of drug administration	34.385	1	34.385	0.141	0.709	0.002
MPH/Placebo ✻ Prime ✻ MPH dosage group ✻ Order of drug administration	313.281	1	313.281	1.284	0.261	0.018
Residuals	17571.751	72	244.052			
MPH/Placebo ✻ Flanker	480.486	1	480.486	7.883	0.006	0.099

Cases	Sum of Squares	df	Mean Square	F	p	η^2_p
MPH/Placebo ✻ Flanker ✻ MPH dosage group	8.571	1	8.571	0.141	0.709	0.002
MPH/Placebo ✻ Flanker ✻ Order of drug administration	32.199	1	32.199	0.528	0.470	0.007
MPH/Placebo ✻ Flanker ✻ MPH dosage group ✻ Order of drug administration	121.587	1	121.587	1.995	0.162	0.027
Residuals	4388.291	72	60.948			
Prime ✻ Flanker	1059.156	1	1059.156	14.923	< .001	0.172
Prime ✻ Flanker ✻ MPH dosage group	35.337	1	35.337	0.498	0.483	0.007
Prime ✻ Flanker ✻ Order of drug administration	8.260	1	8.260	0.116	0.734	0.002
Prime ✻ Flanker ✻ MPH dosage group ✻ Order of drug administration	1.813	1	1.813	0.026	0.873	3.547×10^{-4}
Residuals	5110.341	72	70.977			
MPH/Placebo ✻ Prime ✻ Flanker	6.604	1	6.604	0.127	0.723	0.002
MPH/Placebo ✻ Prime ✻ Flanker ✻ MPH dosage group	29.598	1	29.598	0.568	0.453	0.008
MPH/Placebo ✻ Prime ✻ Flanker ✻ Order of drug administration	1.022	1	1.022	0.020	0.889	2.726×10^{-4}
MPH/Placebo ✻ Prime ✻ Flanker ✻ MPH dosage group ✻ Order of drug administration	7.732	1	7.732	0.148	0.701	0.002
Residuals	3748.952	72	52.069			

Note. Type III Sum of Squares. Significant effects are highlighted in blue font. The significant effects that are underscored were reported in the main manuscript and further analyzed with post hoc tests. Please note that the decision to not report some of the effects (those are not underscored) was based on either the fact that there were higher-level interactions that comprised additional factors, rendering the insights gained from those lower-level significances irrelevant, or on the fact that those interactions did not comprise the relevant task effect (i.e., the interaction of prime * flanker) or the factor of MPH dosage group, which were central to our research question.

Table S-5: Between subject effects of the ANOVA for hit response times.

Cases	Sum of Squares	df	Mean Square	F	p	η^2_p
MPH dosage group	585.808	1	585.808	0.074	0.786	0.001
Order of drug administration	23747.929	1	23747.929	3.000	0.088	0.040
MPH dosage group ∗ Order of drug administration	3368.857	1	3368.857	0.426	0.516	0.006
Residuals	570030.806	72	7917.095			

Note. Type III Sum of Squares. Significant effects are highlighted in blue font.

Table S-6: Descriptive data for hit response times in milliseconds (ms).

MPH/Placebo	Prime Compatibility	Flanker Congruency	MPH Dosage Group	Order of Drug Administration	N	Mean (ms)	SEM
Placebo	Incompatible	Incongruent	0.25 mg/kg	MPH first	17	425.676	6.670
				MPH second	21	437.294	7.810
			0.50 mg/kg	MPH first	20	422.239	7.852
				MPH second	18	435.337	9.189
		Congruent	0.25 mg/kg	MPH first	17	401.999	6.965
				MPH second	21	408.385	6.298
			0.50 mg/kg	MPH first	20	397.465	6.859
				MPH second	18	410.963	6.994
	Compatible	Incongruent	0.25 mg/kg	MPH first	17	375.769	7.533
				MPH second	21	391.343	9.509
			0.50 mg/kg	MPH first	20	381.281	9.314
				MPH second	18	392.889	12.985
		Congruent	0.25 mg/kg	MPH first	17	358.426	6.669
				MPH second	21	367.696	7.838
			0.50 mg/kg	MPH first	20	362.210	7.522
				MPH second	18	374.080	9.884
MPH	Incompatible	Incongruent	0.25 mg/kg	MPH first	17	422.799	9.946
				MPH second	21	431.883	7.181
			0.50 mg/kg	MPH first	20	406.343	7.683
				MPH second	18	429.113	7.073
		Congruent	0.25 mg/kg	MPH first	17	401.163	9.137
				MPH second	21	406.182	5.443
			0.50 mg/kg	MPH first	20	389.019	7.057
				MPH second	18	407.987	8.346
	Compatible	Incongruent	0.25 mg/kg	MPH first	17	367.781	9.842
				MPH second	21	372.574	9.564
			0.50 mg/kg	MPH first	20	369.426	8.936
				MPH second	18	395.795	10.891
		Congruent	0.25 mg/kg	MPH first	17	352.838	9.345
				MPH second	21	353.645	7.459
			0.50 mg/kg	MPH first	20	356.432	7.373
				MPH second	18	376.391	9.308
